# The Roles of Nicotinamide Adenine Dinucleotide Phosphate Reoxidation and Ammonium Assimilation in the Secretion of Amino Acids as Byproducts of Clostridium thermocellum

**DOI:** 10.1128/aem.01753-22

**Published:** 2023-01-10

**Authors:** Johannes Yayo, Thomas Rydzak, Teun Kuil, Anna Karlsson, Dan J. Harding, Adam M. Guss, Antonius J. A. van Maris

**Affiliations:** a Department of Industrial Biotechnology, School of Engineering Sciences in Chemistry, Biotechnology and Health, KTH Royal Institute of Technology, Stockholm, Sweden; b Biosciences Division, Oak Ridge National Laboratory, Oak Ridge, Tennessee, USA; c Center for Bioenergy Innovation, Oak Ridge National Laboratory, Oak Ridge, Tennessee, USA; d Department of Chemical Engineering, School of Engineering Sciences in Chemistry, Biotechnology and Health, KTH Royal Institute of Technology, Stockholm, Sweden; Kyoto Daigaku

**Keywords:** *Acetivibrio thermocellus*, *Clostridium thermocellum*, amino acids, ammonium assimilation, chemostat cultures, glutamate synthase, malate shunt, redox-cofactors

## Abstract

Clostridium thermocellum is a cellulolytic thermophile that is considered for the consolidated bioprocessing of lignocellulose to ethanol. Improvements in ethanol yield are required for industrial implementation, but the incompletely understood causes of amino acid secretion impede progress. In this study, amino acid secretion was investigated via gene deletions in ammonium-regulated, nicotinamide adenine dinucleotide phosphate (NADPH)-supplying and NADPH-consuming pathways as well as via physiological characterization in cellobiose-limited or ammonium-limited chemostats. First, the contribution of the NADPH-supplying malate shunt was studied with strains using either the NADPH-yielding malate shunt (Δ*ppdk*) or a redox-independent conversion of PEP to pyruvate (Δ*ppdk* Δ*malE::P_eno_-pyk*). In the latter, branched-chain amino acids, especially valine, were significantly reduced, whereas the ethanol yield increased from 46 to 60%, suggesting that the secretion of these amino acids balances the NADPH surplus from the malate shunt. The unchanged amino acid secretion in Δ*ppdk* falsified a previous hypothesis on an ammonium-regulated PEP-to-pyruvate flux redistribution. The possible involvement of another NADPH-supplier, namely, NADH-dependent reduced ferredoxin:NADP^+^ oxidoreductase (*nfnAB*), was also excluded. Finally, the deletion of glutamate synthase (*gogat*) in ammonium assimilation resulted in the upregulation of NADPH-linked glutamate dehydrogenase activity and decreased amino acid yields. Since *gogat* in *C. thermocellum* is putatively annotated as ferredoxin-linked, a claim which is supported by the product redistribution observed in this study, this deletion likely replaced ferredoxin with NADPH in ammonium assimilation. Overall, these findings indicate that a need to reoxidize NADPH is driving the observed amino acid secretion, likely at the expense of the NADH needed for ethanol formation. This suggests that metabolic engineering strategies that simplify the redox metabolism and ammonium assimilation can contribute to increased ethanol yields.

**IMPORTANCE** Improving the ethanol yield of *C. thermocellum* is important for the industrial implementation of this microorganism in consolidated bioprocessing. A central role of NADPH in driving amino acid byproduct formation was demonstrated by eliminating the NADPH-supplying malate shunt and separately by changing the cofactor specificity in ammonium assimilation. With amino acid secretion diverting carbon and electrons away from ethanol, these insights are important for further metabolic engineering to reach industrial requirements on ethanol yield. This study also provides chemostat data that are relevant for training genome-scale metabolic models and for improving the validity of their predictions, especially considering the reduced degree-of-freedom in the redox metabolism of the strains generated here. In addition, this study advances the fundamental understanding on the mechanisms underlying amino acid secretion in cellulolytic Clostridia as well as on the regulation and cofactor specificity in ammonium assimilation. Together, these efforts aid in the development of *C. thermocellum* for the sustainable consolidated bioprocessing of lignocellulose to ethanol with minimal pretreatment.

## INTRODUCTION

Clostridium thermocellum is a promising candidate for cost-competitive cellulosic ethanol production through consolidated bioprocessing due to its native ability to efficiently solubilize lignocellulose (1). This anaerobic thermophile (also called Ruminiclostridium thermocellum, Hungateiclostridium thermocellum, and Acetivibrio thermocellus [2]) outperforms other cellulosic microorganisms as well as fungal cellulases in lignocellulose solubilization (3). Recently, this microorganism has been considered for the hybrid biological/catalytic conversion of cellulosic biomass to fuels, in which the ethanol produced by *C. thermocellum* is chemically upgraded to larger fuel molecules that are compatible with heavy-duty, difficult-to-electrify transportation modes (4). However, the industrial implementation of *C. thermocellum* would require improvements of the hitherto achieved ethanol titer (30 g L^−1^ [5]) and yield (75% of the theoretical maximum [6]) to >40 g L^−1^ at >90% of the theoretical maximum yield (4). An increased understanding of the complex and flexible central metabolism of *C. thermocellum* would directly benefit the design-build-test-learn cycles that are aiming to reach these targets.

The secretion of amino acids as byproducts is an atypical phenomenon that is observed in both wild-type and engineered *C. thermocellum* strains, and it diverts sugar away from ethanol formation. Amino acid secretion occurs at both low (5 g L^−1^) and high (93 g L^−1^) loadings of cellulose in batch cultures, in which up to 8.7% of the total consumed carbon is secreted as amino acids, mainly valine (up to 6.0% of the total carbon) and alanine (up to 1.6% of the total carbon) (7–9). In strains engineered and evolved for high ethanol titers (22 g L^−1^) and yields (75% of the theoretical maximum) on cellulose, amino acid secretion increases to 10% of the consumed carbon, becoming the second most abundant organic product, after ethanol (6). Redirecting this carbon and the associated available electrons from amino acids to ethanol will, especially in view of the low profit margins for the production of commodity chemicals and fuels, have a large impact on the economic viability of *C. thermocellum*-based ethanol production.

**FIG 1:**
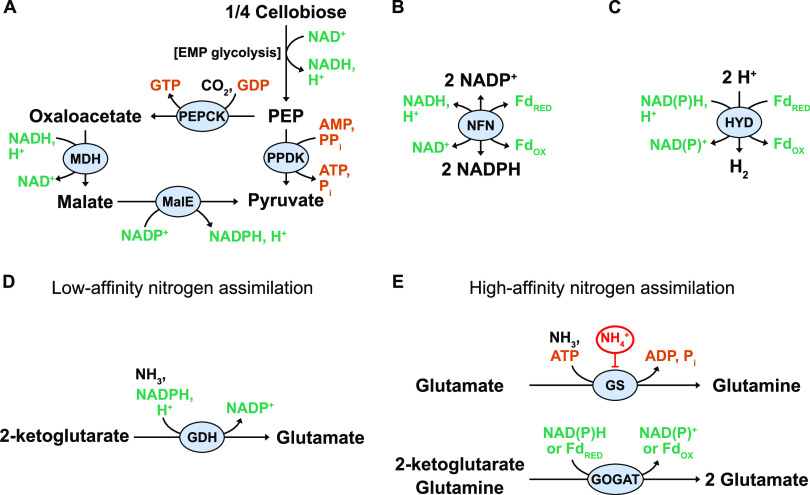
Cofactor usage of *C. thermocellum* in (A) the PEP-to-pyruvate conversion, either via the redox-independent PPDK or via the NADPH-producing malate shunt, (B) the NADH-dependent NFN reaction, (C) a summarized HYD reaction, (D) the low-affinity GDH reaction, and (E) the high-affinity GS-GOGAT cycle. EMP, Embden-Meyerhof-Parnas; PEP, phosphoenolpyruvate; PPDK, pyruvate phosphate dikinase; PEPCK, PEP carboxykinase; MDH, malate dehydrogenase; MalE, malic enzyme; NFN, NADH-dependent reduced ferredoxin:NADP^+^ oxidoreductase; HYD, hydrogenase; GDH, glutamate dehydrogenase; GS, glutamine synthetase; GOGAT, glutamate synthase.

When investigating hypotheses for the observed amino-acid secretion by *C. thermocellum*, it is important to consider how conditions, substrate availability, and metabolic fluxes deviate between laboratory or industrial applications and its natural environment (10, 11). Additionally, it is important to consider the metabolic impact of free-energy conserving mechanisms in its close-to-equilibrium glycolysis (12–14). In one hypothesis, the high nitrogen content in laboratory batch media is thought to result in the accumulation of amino acids (8). Based on this, it was expected that amino acid secretion would be lower in nitrogen-limited conditions. Surprisingly, a study by Holwerda et al. (15) showed the opposite effect in that nitrogen-limited chemostats resulted in a 6-fold higher production rate of secreted amino acids than did carbon-limited chemostats. Specifically, valine was the most abundant, displaying a 20-fold increase, and this was followed by an 8-fold increase in isoleucine and a coinciding 50-fold higher pyruvate secretion. Cell lysis was excluded as a major mechanism, based on calculations on the amino acid distribution in the protein fraction of the biomass as well as on the observed concentrations and patterns in the extracellular amino acids (15). The observation of increased amino acid formation under nitrogen limitation at otherwise identical growth rates in chemostats also makes it unlikely that the increased reversibility of the tRNA charging in protein synthesis, which is caused by *C. thermocellum* lacking a cytosolic pyrophosphatase, is a major contributor to amino acid secretion (15). A third hypothesis by van der Veen et al. (7) proposes that amino acid secretion in *C. thermocellum* is related to redox-cofactor balancing. Investigating this hypothesis requires an understanding of sources and sinks, especially those of NADPH, and of the regulation of the involved pathways by, among other regulators, ammonium. Underlying the hypothesis of van der Veen et al. (7) is the reoxidation of NADPH in amino acid biosynthesis. Based on a current omics-based genome-scale metabolic model (GEM) of *C. thermocellum* (16) and on extrapolation from model microorganisms, the biosynthetic pathways of amino acids from their central building block are expected to predominantly use NADPH (Table 1). For instance, the production of valine from two pyruvate requires two NADPH (Table 1). When considering this possible role of amino acid secretion as an NADPH sink, it is important to note that in *C. thermocellum*, NADPH can additionally be reoxidized through hydrogen formation (Fig. 1C) (17, 18). However, in pure culture laboratory or industrial applications, hydrogen formation has severe thermodynamic limitations, whereas in a natural environment, this is often dealt with through an interspecies hydrogen transfer to methanogens (11, 19, 20).

**TABLE 1:** Precursors and stoichiometric coefficients for cofactors used in amino acid synthesis from precursors in *C. thermocellum*, based on the genome-scale metabolic model iCBI655 (16)^*a*^

Amino acid	Precursor	NH_4_^+^	NADPH	NADH	Fd_reduced_	ATP
Alanine	1 pyruvate	−1	−1 (0)	0 (0)	0 (−1)	0 (0)
Arginine	1 2-ketoglutarate, 1 oxaloacetate	−4	−4 (−1)	0 (0)	0 (−3)	−5 (−8)
Asparagine	1 oxaloacetate	−2	−1 (0)	0 (0)	0 (−1)	−2 (−3)
Aspartate	1 oxaloacetate	−1	−1 (0)	0 (0)	0 (−1)	0 (−1)
Cysteine	1 3-phosphoglycerate, 1 AcCoA	−1	−5 (−4)	1 (1)	0 (−1)	−2 (−3)
Glutamine	1 2-ketoglutarate	−2	−1 (0)	0 (0)	0 (−1)	−1 (−2)
Glycine	1 3-phosphoglycerate	−1	0 (1)	1 (1)	0 (−1)	1 (0)
Glutamate	1 2-ketoglutarate	−1	−1 (0)	0 (0)	0 (−1)	0 (−1)
Histidine	1 ribose 5-phosphate	2	0 (0)	1 (1)	0 (0)	4 (4)
Isoleucine	2 pyruvate, 1 AcCoA	−1	−2 (−1)	1 (1)	0 (−1)	0 (−1)
Leucine	2 pyruvate, 1 AcCoA	−1	−2 (−1)	1 (1)	0 (−1)	0 (−1)
Lysine	1 oxaloacetate, 1 pyruvate	−2	−3 (−1)	−1 (−1)	0 (−2)	0 (−3)
Methionine	1 oxaloacetate, 1 AcCoA	−1	−8 (−7)	−1 (−1)	0 (−1)	−4 (−5)
Phenylalanine	2 PEP, 1 erythrose 4-phosphate	−1	−2 (−1)	0 (0)	0 (−1)	−1 (−2)
Proline	1 2-ketoglutarate	−1	−2 (−1)	−1 (−1)	0 (−1)	−1 (−2)
Serine	1 3-phosphoglycerate	−1	−1 (0)	1 (1)	0 (−1)	0 (−1)
Threonine	1 oxaloacetate	−1	−2 (−1)	−1 (−1)	0 (−1)	−2 (−3)
Tryptophan	2 PEP, 1 erythrose 4-phosphate, 1 3-phosphoglycerate, 1 ribose 5-phosphate	−2	−2 (−1)	1 (1)	0 (−1)	−3 (−4)
Tyrosine	2 PEP, 1 erythrose 4-phosphate	−1	−2 (−1)	1 (1)	0 (−1)	−1 (−2)
Valine	2 pyruvate	−1	−2 (−1)	0 (0)	0 (−1)	0 (−1)

a The numbers in parentheses represent cofactor usage if glutamate production occurred via a ferredoxin-dependent and ATP-dependent glutamine synthetase (GS)-glutamate synthase (GOGAT) cycle instead of a NADPH-dependent glutamate dehydrogenase (GDH).

In contrast to many model heterotrophic microorganisms, in which the pentose phosphate pathway provides most of the NADPH, *C. thermocellum* predominantly uses the malate shunt to supply NADPH by converting phosphoenolpyruvate (PEP) to pyruvate through PEP carboxykinase (PEPCK), NADH-dependent malate dehydrogenase (MDH), and NADP^+^-dependent malic enzyme (encoded by *malE*, Clo1313_1879) (Fig. 1A) (13, 14, 21, 22). An alternative to the malate shunt, namely, PEP-to-pyruvate conversion, can occur through the alternative non-NADPH-producing (redox-independent) pyruvate phosphate dikinase (encoded by *ppdk*, Clo1313_0949) (Fig. 1A). By changing the flux distribution through the malate shunt with flux through Ppdk, more or less NADPH can be formed. Interestingly, both MalE and Ppdk are activated by ammonium, but these activations occur with different activation constants (K_a_). MalE shows a K_a_ for ammonium of 0.7 to 0.8 mM (14, 23), whereas Ppdk requires a higher ammonium concentration, with an estimated K_a_ of 3.8 mM (15). Hence, it has been suggested that at lower intracellular ammonium levels, as is likely under nitrogen limitation, a flux redistribution between Ppdk and MalE that favors NADPH formation may occur (15). If this flux distribution results in an oversupply of NADPH, the increased production of amino acids, such as valine, could act as a NADPH sink. *C. thermocellum* also contains an NADH-dependent reduced ferredoxin:NADP^+^ oxidoreductase (encoded by *nfnAB*, Clo1313_1848-1849) (Fig. 1B) that might contribute to the formation of NADPH by transferring electrons from NADH and reduced ferredoxin to NADP^+^ or, alternatively, reoxidize excess NADPH by producing reduced-ferredoxin and NADH (17).

In addition to the regulatory role on Ppdk and MalE, (intracellular) ammonium levels commonly regulate the activities of the ammonium-assimilation pathways through either glutamate dehydrogenase (GDH) or the higher-affinity glutamine synthetase (GS)-glutamate synthase (GOGAT) (Fig. 1D and E) (24, 25). *C. thermocellum* has genes encoding one NADPH-dependent GDH (Clo1313_1847), four GS (three Type III GS, *glnN*, Clo1313_1357, 2038, 2303; and one Type Iα GS, *glnA*, Clo1313_2031), and a GOGAT cluster (*gogat*, Clo1313_2032-2036) (26, 27). The enzyme activities for GDH and GS were confirmed *in vitro* (15, 28). In line with expectations, GS activity in *C. thermocellum* increases under nitrogen-depleted conditions (28). Despite several attempts, GOGAT activity has hitherto not been measured in *C. thermocellum* (28, 29). Failure to measure NADH- or NADPH-dependent activity is in line with the current KEGG genome annotation of a ferredoxin binding-subunit (Clo1313_2035) in the *gogat* operon of *C. thermocellum* (26, 28, 29). This would mean that a switch from the NADPH-dependent GDH (Fig. 1D) to a higher-affinity ferredoxin-linked GS-GOGAT system (Fig. 1E), would decrease the use of NADPH reoxidized in amino acid biosynthesis (Table 1). In the example of valine, the decrease from two to one NADPH (Table 1) would mean that the regeneration of the same amount of NADPH would require a doubling of the valine secretion flux, possibly contributing to the observed increased amino acid secretion under nitrogen limitation.

The aim of this study was to investigate the role of the NADPH-supplying and NADPH-consuming pathways in *C. thermocellum* on amino acid secretion. The contribution of the NADPH-supplying malate shunt was investigated in strains relying fully on either the NADPH-yielding malate shunt (in a Δ*ppdk* strain) or the redox-independent conversion of PEP-to-pyruvate (in a Δ*ppdk* Δ*malE::P_eno_-pyk* strain). To investigate another potential NADPH source, a Δ*nfnAB* strain was tested under identical conditions. Finally, the role of the GDH/GS-GOGAT node was investigated by testing a Δ*gogat* strain. All strains were characterized and compared to the reference strain in cellobiose-limited or ammonium-limited chemostat cultures through the determination of the substrate, biomass, and extracellular metabolite yields at a fixed dilution rate of 0.1 h^−1^. Enzyme activity assays were performed to confirm the targeted gene deletions as well as to investigate the regulation due to the gene deletions or nutrient limitation.

## RESULTS

### The elimination of the malate shunt decreased amino acid secretion.

The contribution of NADPH formation at the PEP-to-pyruvate node to amino acid secretion was investigated by constructing strains that fully relied on either the NADPH-forming malate shunt or on the alternative redox-independent conversion by Pyk. The removal of *ppdk* resulted in a strain fully relying on the NADPH-forming malate shunt (Δ*ppdk*, named AVM003 [30]). Hitherto, the construction of a strain relying solely on Ppdk for PEP-to-pyruvate conversion has not been successful (13). To obtain a strain fully relying on the redox-independent conversion of PEP-to-pyruvate, the native *malE* gene was swapped with a heterologous pyruvate kinase (*pyk*) from Thermoanaerobacterium saccharolyticum (13, 31) in the Δ*ppdk* strain, resulting in strain AVM064 (Δ*ppdk* Δ*malE::P_eno_-pyk*). Using the Δ*ppdk* background for AVM064 simultaneously avoids competition/interference between Ppdk and Pyk as well as any influence of ammonium activation on Ppdk. The removal of Ppdk and MalE in the respective strains was confirmed via enzyme activity assays, and a high Pyk activity was measured in AVM064 (Table 2). The reference strain showed little background Pyk activity that can be attributed to the combined reaction of PEPCK (13) and MDH (Table 2; Fig. 1) (32).

**TABLE 2:** Activities of Ppdk, MalE, and heterologously expressed Pyk (*T. saccharolyticum*) in batch cultures^*a*^^,^^*b*^

Strain	Enzyme activity (μmol mg_protein_^−1^ min^−1^)		
	Ppdk	MalE	Pyk
Reference strain (DSM 1313)	0.28 ± 0.02	5.8 ± 0.3	0.08 ± 0.03
AVM003 (Δ*ppdk*)	<0.05	12.5 ± 1.3	0.06 ± 0.03
AVM064 (Δ*ppdk* Δ*malE*::P_eno_-*pyk*)	<0.05	<0.05	38.4 ± 2.2

a The detection limit was 0.05 μmol mg_protein_^−1 ^min^−1^.

b Data are shown as the average ± standard deviation for four technical replicates of biological duplicates.

The hypothesis that changes in the flux distribution at the PEP-to-pyruvate node that are caused by various intracellular ammonium concentrations influence amino acid secretion was investigated using the engineered and wild-type strains in chemostat cultures that were limited in either ammonium (N-source) or cellobiose (C-source). If the previously observed increased amino acid secretion by the wild-type under nitrogen limitation (15) is indeed caused by higher activity and a larger flux distribution through the malate shunt, compared to Ppdk, thereby resulting in an NADPH oversupply, amino acid secretion should be high and independent of the nutrient limitation in the strain solely relying on the malate shunt, and amino acid secretion should be constitutively low in the strain solely relying on pyruvate kinase. The chemostats were designed with a feed containing 5 g L^−1^ cellobiose and either 0.09 or 0.7 g L^−1^ ammonium to achieve ammonium-limitation or cellobiose-limitation, respectively, based on the rigorous study by Holwerda et al. (15) that evaluated different C/N ratios. The targeted limitation was confirmed via residual cellobiose and ammonium, respectively, being below the detection limit when the respective substrate was limited and in excess when not limited (Table 3). As commonly seen for various microorganisms when growth is not limited by the energy source (31, 33–35), the ammonium-limited chemostats showed higher cellobiose uptake rates and slightly lower biomass yields on cellobiose (Table 3).

**TABLE 3:** Physiological parameters in chemostats with either cellobiose or ammonium limitation, at a dilution rate of 0.10 h^−1^^*a*^^,^^*b*^

Parameter	Cellobiose limitation					Ammonium limitation				
	Reference strain (LL345)	AVM064 (Δ*ppdk* Δ*malE*::p*_eno_*-*pyk*)	AVM003 (Δ*ppdk*)	LL1084 (Δ*nfnAB*)	AG1715 (Δ*gogat*)	Reference strain (LL345)	AVM064 (Δ*ppdk* Δ*malE*::p*_eno_*-*pyk*)	AVM003 (Δ*ppdk*)	LL1084 (Δ*nfnAB*)	AG1715 (Δ*gogat*)
Cellobiose in feed (g L^−1^)	4.70 ± 0.03	4.98 ± 0.03	4.76 ± 0.08	4.87 ± 0.02	4.80 ± 0.04	4.69 ± 0.04	4.99 ± 0.00	4.63 ± 0.04	4.85 ± 0.02	4.77 ± 0.06
Residual cellobiose (g L^−1^)	<0.01	<0.01	<0.01	<0.01	<0.01	1.83 ± 0.03	1.10 ± 0.04	1.66 ± 0.11	1.57 ± 0.10	2.23 ± 0.30
Ammonium in feed (g L^−1^)	0.69 ± 0.00	0.71 ± 0.02	0.67 ± 0.02	0.70 ± 0.01	0.70 ± 0.01	0.087 ± 0.003	0.089 ± 0.001	0.087 ± 0.001	0.089 ± 0.001	0.089 ± 0.001
Residual ammonium (g L^−1^)	0.51 ± 0.01	0.53 ± 0.01	0.50 ± 0.01	0.45 ± 0.01	0.45 ± 0.01	<0.002	<0.002	<0.002	<0.002	<0.002
q_Cellobiose_ (mmol g_biomass_^−1^ h^−1^)	−2.19 ± 0.03	−2.36 ± 0.08	−2.07 ± 0.09	−2.20 ± 0.02	−2.15 ± 0.02	−2.49 ± 0.02	−2.54 ± 0.10	−2.40 ± 0.10	−2.45 ± 0.12	−2.11 ± 0.06
q_Ammonium_ (mmol g_biomass_^−1^ h^−1^)	−1.60 ± 0.04	−1.62 ± 0.07	−1.56 ± 0.04	−2.14 ± 0.12	−2.07 ± 0.06	−1.43 ± 0.05	−1.10 ± 0.05	−1.33 ± 0.10	−1.26 ± 0.03	−1.41 ± 0.11
Biomass yield (g g_cellobiose_^−1^)	0.13 ± 0.00	0.12 ± 0.01	0.15 ± 0.01	0.13 ± 0.00	0.14 ± 0.00	0.12 ± 0.00	0.11 ± 0.00	0.13 ± 0.01	0.12 ± 0.01	0.14 ± 0.00
Glycogen content (%, g g_biomass_^−1^)	1.1 ± 0.6	ND^*g*^	ND	ND	ND	31.5 ± 0.6	ND	ND	ND	ND
Ethanol yield (mol mol_cellobiose_^−1^)	0.97 ± 0.06	1.55 ± 0.01	0.96 ± 0.03	0.96 ± 0.02	0.84 ± 0.07	1.07 ± 0.03	1.55 ± 0.05	0.84 ± 0.05	1.00 ± 0.02	0.77 ± 0.03
Acetate yield (mol mol_cellobiose_^−1^)	1.38 ± 0.01	0.65 ± 0.02	1.46 ± 0.04	1.38 ± 0.02	1.38 ± 0.01	0.94 ± 0.01	0.61 ± 0.01	1.17 ± 0.12	1.01 ± 0.01	0.84 ± 0.07
Formate yield (mol mol_cellobiose_^−1^)	0.11 ± 0.04	0.22 ± 0.02	0.10 ± 0.01	0.31 ± 0.01	0.07 ± 0.01	0.15 ± 0.01	0.32 ± 0.03	0.18 ± 0.05	0.13 ± 0.03	0.30 ± 0.12
Lactate yield (mol mol_cellobiose_^−1^)	0.01 ± 0.00	0.02 ± 0.00	0.02 ± 0.00	0.01 ± 0.00	0.02 ± 0.00	0.07 ± 0.01	0.04 ± 0.02	0.06 ± 0.02	0.06 ± 0.00	0.04 ± 0.02
Pyruvate yield (mol mol_cellobiose_^−1^)	<0.01	<0.01	0.01 ± 0.01	<0.01	0.01 ± 0.00	0.09 ± 0.00	0.01 ± 0.00	0.02 ± 0.00	0.03 ± 0.00	0.02 ± 0.01
Malate yield (mol mol_cellobiose_^−1^)	<0.01	0.11 ± 0.02	<0.01	<0.01	<0.01	<0.01	0.03 ± 0.00	<0.01	<0.01	<0.01
Total amino acids yield (mol mol_cellobiose_^−1^)^*c*^	0.026 ± 0.001	0.031 ± 0.002	0.028 ± 0.002	0.030 ± 0.001	0.022 ± 0.001	0.116 ± 0.002	0.044 ± 0.006	0.107 ± 0.026	0.115 ± 0.009	0.038 ± 0.010
CO_2_ yield (mol mol_cellobiose_^−1^)^*d*^	2.38 ± 0.02	2.00 ± 0.04	2.48 ± 0.06	2.18 ± 0.03	2.29 ± 0.05	2.07 ± 0.03	1.96 ± 0.01	2.06 ± 0.08	2.11 ± 0.02	1.48 ± 0.12
H_2_ yield (mol mol_cellobiose_^−1^)	4.85 ± 0.06	ND	ND	ND	4.73 ± 0.05	2.44 ± 0.09	ND	ND	ND	4.26 ± 0.46
Ex. protein yield (mol mol_cellobiose_^−1^)^*e*^	0.09 ± 0.00	0.06 ± 0.00	0.08 ± 0.00	0.10 ± 0.00	0.08 ± 0.00	0.05 ± 0.00	0.04 ± 0.00	0.05 ± 0.00	0.04 ± 0.00	0.04 ± 0.01
Carbon recovery (%)^*f*^	81 ± 1	77 ± 0	84 ± 2	81 ± 1	77 ± 1	77 ± 1	75 ± 1	77 ± 3	76 ± 1	64 ± 1
Nitrogen recovery (%)^*f*^	71 ± 2	66 ± 2	70 ± 2	54 ± 3	53 ± 2	87 ± 2	95 ± 3	96 ± 2	97 ± 1	72 ± 6
Degree-of-reduction recovery (%)^*f*^	70 ± 1	73 ± 0	72 ± 2	70 ± 1	66 ± 1	69 ± 1	71 ± 1	68 ± 2	68 ± 1	59 ± 1
Degree-of-reduction recovery with H_2_ (%)^*f*^	90 ± 1	ND	ND	ND	86 ± 2	79 ± 1	ND	ND	ND	76 ± 1

a Data are shown as the average ± standard deviation for three biological replicates.

b Negative values represent consumption, and positive values represent production.

c Excluding cysteine.

d Calculated from the fermentation product yields and biomass yield (see Materials and Methods).

e Based on the molecular weight of a generalized protein composition (see Table S1 and Materials and Methods).

f Calculated as the ratio of the total element or degree-of-reduction found in the products to the total element or degree-of-reduction in the consumed substrate (Tables S3–S5).

g ND, not determined.

**FIG 2:**
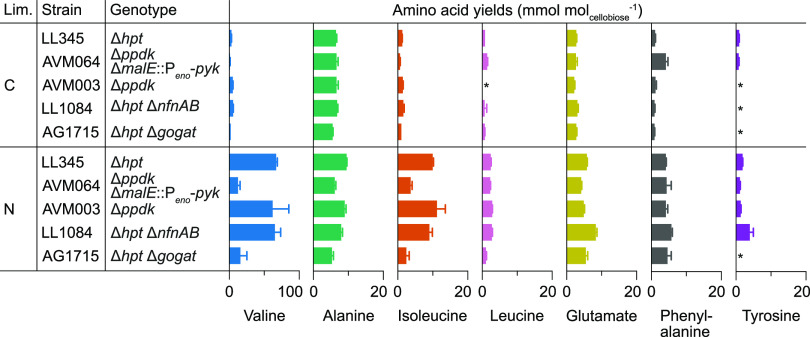
Amino acid yields in chemostats with either cellobiose (C) or ammonium (N) as the sole limiting nutrient at a dilution rate of 0.10 h^−1^. The remaining amino acids can be found in Table S6 and were either below the detection limit (*, <0.5 mmol mol_cellobiose_^−1^) or only showed small changes. The error bars represent the standard deviation of three biological replicates.

To create a baseline for the subsequent comparisons of the other strains under identical conditions, the reference strain LL345 was grown in cellobiose-limited or ammonium-limited chemostats (Table 3). The total amino acid yield increased 4.5-fold, from 0.026 ± 0.001 mol mol_cellobiose_^−1^ under cellobiose limitation to 0.116 ± 0.002 mol mol_cellobiose_^−1^ under ammonium limitation (Table 3). Not only did the yields of the pyruvate-derived amino acids alanine (1.5-fold), isoleucine (7.8-fold), leucine (3.1-fold), and valine (20.4-fold) increase, but the extracellular pyruvate (from <0.01 to 0.09 ± 0.00 mmol mol_cellobiose_^−1^) and lactate yields (4.7-fold) also increased (Table 3; Fig. 2; Table S2). Other amino acid yields that increased were methionine (from undetected to 6.3 ± 0.5 μmol mol_cellobiose_^−1^), phenylalanine (3.9-fold), glutamate (2.1-fold), and tyrosine (2.1-fold) (Fig. 2; Table S2). No significant changes were observed for asparagine, aspartate, glutamine, arginine, glycine, histidine, threonine, or tryptophan secretion, whereas the serine yield dropped by 26% (Table S2). In absolute numbers, valine, alanine, and glutamate each accounted for approximately 15 to 20% of the secreted amino acids under cellobiose limitation (Fig. 2). Under ammonium limitation, valine was by far the most abundant, at 58% of the secreted amino acids (Fig. 2). As a result, secreted amino acids accounted for 4.9% of the carbon and 5.7% of the degree-of-reduction of the consumed cellobiose under ammonium limitation, compared to only 0.9% and 0.9%, respectively, under cellobiose limitation (Table S3 and S4). Similarly, 20.3% of the total consumed nitrogen ended up in secreted amino acids under ammonium limitation, compared to 3.7% under cellobiose limitation (Table S5). Among the traditional fermentation products, the largest change was observed for the H_2_ yield, which dropped by 50% under ammonium limitation. Additionally, the intracellular glycogen content for the reference strain increased from 1.1 ± 0.6% (wt/wt) under cellobiose limitation to 31.5 ± 0.6% (wt/wt) under ammonium limitation. The results of this baseline characterization of the reference strain in cellobiose-limited/ammonium-limited chemostats are consistent with the previous results of Holwerda et al. (15).

Under identical conditions, the strain fully relying on Pyk for the conversion of PEP-to-pyruvate (AVM064; Δ*ppdk* Δ*malE::P_eno_-pyk*), and thereby lacking the NADPH provided by the malate shunt, only showed a 1.4-fold increase (*P < *0.05) in the total amino acid yield from cellobiose limitation to ammonium limitation, compared to the 4.5-fold increase observed for the reference strain (Table 3). Although valine secretion increased 10-fold (*P < *0.05) from cellobiose to ammonium limitation, the valine yield under ammonium limitation was still 81% lower than that of the reference strain (Fig. 2; Table S2). Increases in the glutamate (1.6-fold), isoleucine (5.6-fold), leucine (1.6-fold), and tyrosine (1.4-fold) yields (*P < *0.05) under ammonium limitation were also lower than that of the reference strain by 28%, 63%, 8%, and 40%, respectively (*P < *0.05) (Fig. 2). No significant differences between the two limitations for AVM064 were observed for alanine and lactate (Fig. 2), whereas pyruvate increased from <0.01 to 0.01 ± 0.00 mol mol_cellobiose_^−1^ (*P < *0.05), which was 87% less than that of the reference strain under ammonium limitation (Table 3). Overall, rerouting the PEP-to-pyruvate conversion through Pyk rather than through the malate shunt decreased the total carbon, degree-of-reduction, and nitrogen ending up in the secreted amino acids under nitrogen limitation to 1.9%, 2.1%, and 10.8%, respectively, compared to 4.9%, 5.7%, and 20.3% for the reference strain under the same conditions (Tables S3–S5). The malate shunt in AVM064 was interrupted through the removal of malic enzyme while maintaining malate dehydrogenase, resulting in the secretion of malate with a yield of 0.11 ± 0.02 mol mol_cellobiose_^−1^ under cellobiose limitation and 0.03 ± 0.00 mol mol_cellobiose_^−1^ under ammonium limitation (Table 3). In line with a switch from the malate shunt, which transfers electrons from NADH to NADP^+^, to the redox-independent PEP-to-pyruvate conversion in the Pyk-dependent strain, the ethanol yield increased by 46 to 60% to 1.55 mol mol_cellobiose_^−1^ under either nutrient limitation, compared to the reference strain (Table 3).

The physiological impact of deleting *ppdk*, which thereby routed the entire PEP-to-pyruvate conversion through the NADPH-yielding malate shunt, did not result in any drastic changes in product yields, compared to the reference strain (Table 3). In the ammonium-limited chemostat cultures, the impact of *ppdk* deletion was slightly higher, with a 21% decrease in the ethanol yield, from 1.07 ± 0.03 mol mol_cellobiose_^−1^ in the reference strain to 0.84 ± 0.05 mol mol_cellobiose_^−1^ in the Δ*ppdk* strain, and a corresponding increase in the acetate yield (Table 3). The amino acid yields under both limitations were similar to those of the reference strain (Fig. 2; Table S2).

The lack of a physiological impact of the *ppdk* deletion in the cellobiose-limited chemostats (Table 3) suggests that the flux through Ppdk in the wild-type under this condition was likely insignificant. Even though NADPH supply through the malate shunt is clearly required for surplus amino acid secretion under ammonium-limited conditions, as is seen from the strongly decreased total amino acid yield observed for AVM064 (Δ*ppdk* Δ*malE*::P*_eno_*-*pyk*) (Table 3), this suggests a more complex underlying mechanism than only the ammonium-dependent redistribution of the PEP-to-pyruvate conversion fluxes between Ppdk and MalE.

### No impact of *nfnAB* deletion on amino acid secretion.

To investigate the role of the NADH-dependent reduced ferredoxin:NADP^+^ oxidoreductase (NfnAB) (Fig. 1B) in amino acid secretion, a Δ*nfnAB* strain (LL1084) (17) was characterized in cellobiose-limited or ammonium-limited chemostats. A comparison of this strain with the reference strain in cellobiose-limited chemostats showed the same biomass yield and cellobiose uptake rate under both conditions (Table 3). Under cellobiose limitation, there was a 2.7-fold increase (*P < *0.05) in the formate yield, compared to that of the reference strain at identical acetate and ethanol yields (Table 3). This suggests a small shift from pyruvate ferredoxin oxidoreductase (PFOR), which reduces ferredoxin, to pyruvate formate-lyase (PFL) in the Δ*nfnAB* strain. Interestingly, under ammonium limitation, the formate yield was the same as that of the reference strain, suggesting a negligible flux through NfnAB in the reference strain under this condition or the full functional complementation by the other NADPH-supplying routes. In line with this, the deletion of *nfnAB* also did not result in a meaningful change in the total amino acid formation (Fig. 2). Similar to the cellobiose-limited chemostats, the ammonium limitation of the Δ*nfnAB* strain showed the same biomass yield and cellobiose uptake rate as did the reference strain (Table 3). Only a minor decrease of the pyruvate yield from 0.09 ± 0.00 mol mol_cellobiose_^−1^ in the reference strain to 0.03 ± 0.00 mol mol_cellobiose_^−1^ in the Δ*nfnAB* strain, as well as an accompanying increase of the acetate yield by the same magnitude (*P < *0.05), were observed.

### The deletion of *gogat* decreased amino acid secretion under nitrogen limitation.

To investigate whether GOGAT upregulation and the predicted accompanying shift from NADPH-dependent to partially ferredoxin-linked ammonium assimilation contribute to amino acid secretion under nitrogen limitation, a Δ*gogat* strain (AG1715) was constructed and characterized in cellobiose-limited or ammonium-limited chemostats. Under cellobiose limitation, in which both strains showed high NADPH-linked GDH activities (25.0 ± 2.5 and 32.3 ± 1.3 μmol mg_protein_^−1 ^min^−1^) (Table 4) and in which GDH is expected to carry the majority of the ammonium-fixation flux, the Δ*gogat* strain and the reference strain showed the same yields and rates for canonical fermentation products (Table 3). Under these conditions, a small decrease of 15% (*P < *0.05) was observed in total amino acid secretion in the Δ*gogat* strain, compared to the reference strain (Table 3).

**TABLE 4:** Glutamate dehydrogenase activities in LL345 and AG1715 from steady-state cultures grown in cellobiose-limited or ammonium-limited chemostats, expressed in μmol mg_protein_^−1^ min^−1^^*a*^

GDH-	Cellobiose limitation		Ammonium limitation	
	Reference strain (LL345)	AG1715 (Δ*gogat*)	Reference strain (LL345)	AG1715 (Δ*gogat*)
NADPH	25.0 ± 2.5	32.3 ± 1.3	0.39 ± 0.04	62.3 ± 10.3
NADH	0.16 ± 0.01	0.38 ± 0.03	<0.05	0.36 ± 0.02

a Data are shown as the average ± standard deviation for four technical replicates of biological duplicates.

In ammonium-limited chemostats, the GDH activity of the reference strain dramatically decreased 64-fold to 0.39 ± 0.04 μmol mg_protein_^−1 ^min^−1^ (Table 4), which is in line with a textbook switch to the higher-affinity GS-GOGAT system (activity not measured). With GDH being the sole ammonium-assimilating option in the Δ*gogat* strain, GDH activity was upregulated 160-fold to 62.3 ± 10.3 μmol mg_protein_^−1 ^min^−1^, compared to the reference strain under ammonium limitation (Table 4). Interestingly, comparing the Δ*gogat* strain between growth conditions also showed a 2-fold upregulation (*P < *0.05) of GDH activity under ammonium limitation. This upregulation was likely needed to compensate for the (predicted) lower intracellular ammonium concentration and to maintain the same ammonium-assimilation flux that was needed at the identical growth rates of 0.10 h^−1^.

In the ammonium-limited chemostats, the total amino acid secretion decreased by 67%, from 0.116 ± 0.002 mol mol_cellobiose_^−1^ in the reference strain to 0.038 ± 0.010 mol mol_cellobiose_^−1^ in the Δ*gogat* strain (Table 3). Valine decreased the most and had a yield of 16.0 ± 9.4 mmol mol_cellobiose_^−1^ under ammonium limitation, which was 76% lower than that of the reference strain (*P < *0.05) (Fig. 2). The yields of other pyruvate-derived amino acids also significantly decreased, compared to the reference strain, with 44% lower alanine, 75% lower isoleucine, and 56% lower leucine under ammonium limitation (*P < *0.05) (Fig. 2). All remaining amino acid yields, except that of phenylalanine, were significantly lower in the Δ*gogat* strain (*P < *0.05) (Fig. 2; Table S2). The relative abundance of valine dropped from 58% of the total secreted amino acids in the reference strain to 42% in the Δ*gogat* strain (Table S2). Phenylalanine, glutamate, and alanine followed, corresponding to roughly 12%, 15%, and 14%, respectively. Overall, the deletion of *gogat* decreased the amounts of carbon, degree-of-reduction, and nitrogen consumed that were diverted to the amino acid byproducts under ammonium limitation from 4.9%, 5.7%, and 20.3% in the reference strain to 1.6%, 1.8%, and 5.9% in the Δ*gogat* strain (Tables S3–S5).

Surprisingly, the deletion of *gogat* resulted in a large increase of the H_2_ yield under ammonium limitation. The reference strain had a H_2_ yield of 2.44 ± 0.09 mol mol_cellobiose_^−1^, whereas the Δ*gogat* strain showed a H_2_ yield of 4.26 ± 0.46 mol mol_cellobiose_^−1^ (Table 3), which might reflect an increased need for ferredoxin reoxidation through the hydrogenases. Concomitantly, the ethanol yield decreased with 0.3 mol mol_cellobiose_^−1^ (27%) (*P < *0.05), whereas the formate yield doubled to 0.30 ± 0.12 mol mol_cellobiose_^−1^ (*P < *0.05) (Table 3). These changes in the redox metabolism would be in line with the increased stoichiometric need for NADPH in the ammonium assimilation if the NADPH that was formed through the malate shunt were to go at the expense of NADH. The biomass yield increased by 19%, compared to the reference strain, which can (at least, directionally) be explained by the ATP savings made in biosynthesis when shifting from ammonium assimilation via the ATP-consuming GOGAT-GS cycle to the ATP-neutral GDH reaction.

## DISCUSSION

Using targeted gene deletions and cellobiose-limited/ammonium-limited chemostats as diagnostic tools, this study found several indications that amino acid secretion is driven by a cellular need to balance NADPH. This was illustrated by decreased amino acid yields upon the elimination of the NADPH-supplying malate shunt and separately, likely by the changing of the cofactor specificity of ammonium assimilation. Although the hypothesis of the differential regulation of the malate shunt and Ppdk by the ammonium concentration was falsified, the mechanisms underlying amino acid secretion clearly involve an oversupply of NADPH that, due to transhydrogenation in the malate shunt, comes at the expense of the NADH supply. The lower NADH supply and an apparent minimal contribution of NfnAB for NADPH-to-NADH conversion might limit the conversion of pyruvate to lactate and ethanol and instead favor the production of NADPH-linked, pyruvate-derived amino acids. Using amino acid formation to regenerate NADP^+^ has been observed in other species, as well. This mechanism has been proposed in the fungus Aspergillus nidulans (36) and in the alanine-producing archaeon Pyrococcus furiosus (37). Anaerobic rumen bacteria also commonly produce amino acids that regenerate NADP^+^ at a low ATP cost (38). Amino acid secretion is found among other Clostridia species as well (34, 39), which generally have 5 to 20-fold higher intracellular amino acid levels than do Gram-negative bacteria (40).

In addition to the NADPH originating from the malate shunt, specifically under ammonium limitation, a shift from GDH to GOGAT likely decreases the NADPH reoxidized per amino acid and thereby increases amino acid secretion in wild-type *C. thermocellum* under these laboratory conditions. Underlying that hypothesis is the proposed use of ferredoxin as the redox cofactor of GOGAT. This currently unconfirmed annotation (26) is supported by the observed changes in the redox metabolism of the reference strain and Δ*gogat* strain under ammonium limitation (Table 3; Fig. 2). The drop in the H_2_ yield of the reference strain under ammonium limitation (Table 3) is in line with a switch from (mainly) NADPH-dependent GDH activity in cellobiose limitation to (mainly) ferredoxin-linked GS-GOGAT activity in ammonium assimilation, which decreases the availability of reduced ferredoxin for H_2_ formation. In addition to this switch, a 17% decrease in the production of reduced ferredoxin by pyruvate ferredoxin oxidoreductase (PFOR) (Table 3), calculated as $Y_{\mathrm{acetate}/\mathrm{cellobiose}}+Y_{\mathrm{ethanol}/\mathrm{cellobiose}}-Y_{\mathrm{formate}/\mathrm{cellobiose}}$, is likely also contributing to the drop in the H_2_ yield. The Δ*gogat* strain would likely require other mechanisms to reoxidize ferredoxin, which, in *C. thermocellum*, can (in theory) occur through: (i) H_2_ production via a proton-pumping, energy-converting hydrogenase (ECH) or a bifurcating NAD(P)H-hydrogenase; (ii) a proton-pumping ferredoxin:NAD^+^ oxidoreductase (RNF) that increases the NADH pool; (iii) NfnAB, by consuming NADH and producing NADPH; or (iv) a cycle with reverse PFOR and forward PFL that assimilates CO_2_ and generates formate (17). The decreased ethanol yield (suggesting a lower NADH availability), increased formate yield, decreased amino acid yields, and increased H_2_ yield of the Δ*gogat* strain under ammonium limitation suggest that the combination of hydrogenases and a 40% lower net flux through PFOR, calculated as $q_{\mathrm{acetate}}+q_{\mathrm{ethanol}}-q_{\mathrm{formate}}$, (Table S6) replace GOGAT as a ferredoxin sink (Table 3; Fig. 2).

Current stoichiometric metabolic models of *C. thermocellum* contain the “free” shuffling of electrons between NAD(P) and ferredoxin via the transhydrogenases NFN and RNF, which ultimately results in H_2_ instead of amino acids as an electron sink (16, 41). However, the metabolic flux and a thermodynamic analysis suggest a narrow window for thermodynamically favorable conversion via the (trans)hydrogenases (42). The lack of a significant effect on the fermentation product profile of Δ*nfnAB* in chemostat cultures in the present study and in the batch cultures in the study of Lo et al. (17) suggests either a minimal contribution or the full complementarity of NFN activity under the tested conditions in these strain backgrounds. In laboratory environments, H_2_ production via hydrogenases is likely further thermodynamically limited by H_2_ supersaturation due to poor liquid-to-gas transfer (43). Physiological predictions and the design of metabolic engineering strategies would benefit from including these thermodynamic limitations into their models.

The amino acid secretion observed under these laboratory conditions might be less common in the natural environments of cellulolytic Clostridia, as these niches not only are deficient in bioavailable nitrogen but also commonly contain H_2_-consuming methanogens (10, 11). In the presence of these methanogens, hydrogen partial pressures are maintained at low levels, which makes H_2_ production as an electron sink more favorable and increases the energy efficiency in the metabolism (10, 11). Indeed, cocultures with H_2_-consuming methanogens result in higher acetate-to-ethanol ratios (11), whereas increasing the (dissolved) H_2_ partial pressure results in a higher ethanol-to-acetate ratio, a decreased H_2_ yield, and reverse NAD(P)H-hydrogenase activity (44–46). Sparging laboratory cultures with N_2_, which is believed to make H_2_ production more favorable, shifted the fermentation profile, with increases in the ethanol and acetate titers and a decrease in the valine titer (42). On top of thermodynamic limitations, hydrogenases are complex organometallic proteins that require specific systems for posttranslational assembly (18, 47). Hence, their synthesis might consume more ATP than that of proteins involved in the valine and alanine pathways. Such kinetic limitations were observed with Clostridium cellulolyticum, in which chemostat cultures at various dilution rates showed that the regeneration of cofactors through hydrogen gas formation was too slow at high carbon fluxes and resulted in more lactate and polysaccharides (33, 48). Importantly, the diversion of electrons to either hydrogen and/or methane in mixed cultures goes at the expense of the ethanol yield on the substrate, necessitating a choice between lower theoretical ethanol yields or the continued engineering of *C. thermocellum* for improved product yields.

Aiming to get closer to the maximum theoretical ethanol yield would require further metabolic engineering to decrease the 10% of the consumed carbon that is currently diverted to amino acids in the *C. thermocellum* strain with the highest yield (6). One strategy to reduce amino acid secretion would be to simplify the redox metabolism by reducing the diversity in the redox cofactors. Similar to Saccharomyces cerevisiae, which relies solely on NADH for glycolysis and ethanol production, this would likely reduce the NADPH-driven amino acid secretion observed in *C. thermocellum*. As a first step, this study has shown that deleting the NADPH-supplying MalE (Δ*ppdk* Δ*malE*::P_eno_-*pyk*) increased the ethanol and malate yields, whereas the amino acid yields in chemostat cultures dropped, reflecting a higher NADH availability and a lower NADPH availability. This is in line with the results of a study by Olson et al. (32), in which a similarly constructed strain (Δ*ppdk* Δ*malE*-*mdh* Δ*ldh*::P*_eno_*-*pyk*) in batch cultures showed a 3-fold increase in ethanol production, whereas valine production dropped 3-fold. However, engineering of the steps required to convert pyruvate to ethanol in a scenario with a simplified redox metabolism, through either (i) PFOR, AdhE, and RNF or (ii) through a pyruvate decarboxylase (PDC: pyruvate → acetaldehyde + CO_2_) and NADH-dependent alcohol dehydrogenase, has had limited success in achieving a sufficiently high ethanol yield and titer and requires more research (49–51). Alternatively, the ethanol production pathway could be changed to reoxidize the NADPH that is otherwise diverted to amino acid secretion. For instance, the laboratory evolution of a Δ*pta* Δ*ldh* strain resulted in a point mutation in the bifunctional alcohol/aldehyde dehydrogenase (*adhE*) that allowed for the use of NADPH on top of NADH as a cofactor, thereby producing more ethanol and less amino acids (5, 7, 15). Combining the above interventions with a deletion in glutamate synthase and/or *gogat* might further reduce amino acid secretion. In line with that, Rydzak et al. (27) showed that the deletion of the GS gene *glnA* increased the ethanol yield and reduced the secretion of amino acids significantly. Overall, these findings can guide engineering to decrease the amino acid secretion and improve the ethanol yield of *C. thermocellum* for the sustainable, consolidated bioprocessing of lignocellulosic biomass.

## MATERIALS AND METHODS

### Strains and maintenance.

The *C. thermocellum* DSM 1313 wild-type (WT) strain was purchased from the DSMZ microorganism collection (www.dsmz.de). All strains used or constructed in this study are listed in Table 5. Freezer stocks were prepared by growing strains in the complex medium CTFUD (described by Olson and Lynd [52]) at 55°C to an optical density (OD) of 0.6 to 2.0. After the addition of glycerol to 25% (vol/vol), 1 mL aliquots were stocked in cryo-vials (VWR, Stockholm, Sweden) at −80°C. Strain construction and stocking were performed in an anaerobic vinyl chamber from Coy Laboratory Products (TG Instruments, Helsingborg, Sweden) with an atmosphere of 5% H_2_, 10% CO_2_, and 85% N_2_ (Strandmöllen AB, Ljungby, Sweden).

**TABLE 5:** Strains used in this study

Strain name	Parent strain	Genotype	Reference
Wild-type (WT)	WT	DSM 1313	DSMZ^*a*^
LL345 or M1354	WT	DSM 1313 Δ*hpt*	(63)
AVM003	WT	DSM 1313 Δ*ppdk*	(30)
AVM064	AVM003	DSM 1313 Δ*ppdk* Δ*malE::*P*_eno_*-*pyk*^*b*^	This study
LL1084	LL345	DSM 1313 Δ*hpt* Δ*nfnAB*	(17)
AG1715	LL345	DSM 1313 Δ*hpt* Δ*gogat* (Clo1313_2032-2036)	This study

a DSMZ, German Collection of Microorganisms and Cell Cultures GmbH, Braunschweig, Germany (www.dsmz.de).

b The *pyk* gene (*tsac_1363*) is from the Thermoanaerobacterium saccharolyticum JW/SL-YS 485 genome.

### Plasmid and strain construction.

The plasmids and primers used in this study are listed in Tables 5 and 6. Plasmid construction and propagation were performed in Escherichia coli Top10 (*dam*^+^ *dcm*^+^) (Invitrogen, Carlsbad, CA) and BL21 (*dam*^+^ *dcm*^−^) (New England Biolabs, Ipswich, MA) aerobically in LB medium supplemented with 12 to 25 μg mL^−1^ chloramphenicol. The purification of the plasmid DNA, genomic DNA, and PCR products were performed with commercially available kits from GeneJET (Thermo Fisher Scientific) and QIAprep (Qiagen, Germantown, MD). Primers were purchased from Invitrogen (Thermo Fischer Scientific). The plasmids were designed for markerless gene deletion according to the methods of Olson and Lynd (52), using pDGO145 as a backbone (53). Phusion High-Fidelity DNA polymerase (Thermo Scientific) was used to amplify fragments from *C. thermocellum* genomic DNA that corresponded to 500 to 1,000 bp upstream (5′-flank), downstream (3′-flank), and internal (int) regions of the coding sequences of interest (*malE*, Clo1313_1879; *gogat*, Clo1313_2032-2036) as well as to the promoter for the enolase gene (178 bp upstream of Clo1313_2090 on the reverse strand) (54), using the primers listed in Table 7. The *pyk* gene (Tsac_1363) was amplified from the genomic DNA of *T. saccharolyticum* JW/SL-YS 485 (DSM 8691), using primers 0553 and 0564. The fragments were assembled into the backbone according to the Gibson protocol (55), generating plasmids pJY19 (simultaneous deletion of *malE* and insertion of P*_eno_*-*pyk*) and pNJ22::GOGAT_del (Δ*gogat*). For the simultaneous deletion and insertion, the P*_eno_*-*pyk* gene was placed between the 5′-flank and the 3′-flank (56). The plasmids were verified via diagnostic PCR and sequencing. Finally, the propagation of plasmids in E. coli BL21 (*dam*^+^ *dcm*^−^) ensured correct methylation before transformation into *C. thermocellum* (57). The transformation and selection for markerless gene editing in *C. thermocellum* were performed according to the methods of Olson and Lynd in CTFUD medium (52). *malE* was deleted while simultaneously inserting P*_eno_*-*pyk* at the *malE* locus in strain AVM003 (Δ*ppdk*), thereby generating AVM064 (Δ*ppdk* Δ*malE::*P*_eno_*-*pyk*). The *gogat* gene cluster was deleted in LL345 (Δ*hpt*) using pNJ22::GOGAT_del, thereby generating strain LL1715 (Δ*hpt* Δ*gogat*). The sequencing of the target loci and the 16S rRNA locus, using the primers listed in Table 7, was performed to confirm the correctness of the gene edits and the culture purity.

**TABLE 6:** Plasmids used in this study

Plasmid name	Description	Genbank accession no.	Source or reference
pDGO145	Plasmid backbone for deletion/insertion	KY852359	53
pJY19	Simultaneous deletion of *malE* (Clo1313_1879) and insertion of P*_eno_*-*pyk*	OP441060	This study
pNJ22::GOGAT_del	Deletion of *gogat* (Clo1313_2032-2036)	OP441061	This study

**TABLE 7:** Primers used in this study

ID	Description	Sequence (5′ to 3′)^*a*^
0001	16S rRNA fragment to confirm culture purity	ACGGCTACCTTGTTACGACTT
0002	16S rRNA fragment to confirm culture purity	ACGGCTACCTTGTTACGACTT
0224	pDGO145 backbone	GATATCGCCTCGTGATACGC
0225	pDGO145 backbone	CAGCTGCTAATAGTAGTGAAAAAATCAG
0226	P_gapDH_-*cat*-*hpt* selection cassette from pDGO145	CTGAACTACTGGGCCAGGTATG
0227	P_gapDH_-*cat*-*hpt* selection cassette from pDGO145	ATCGTGGGAATAGGCATGG
0557	Internal region of *malE* with an overhang matching the pDGO145 backbone	gcggcattatccctgattttttcactactattagcagctgGTTTATCTGGTTCGGGAAGTCG
0558	Internal region of *malE* with an overhang matching the P*_gapDH_*-*cat*-*hpt* cassette	cctccccatgctttaatacatacctggcccagtagttcagCGAAGATATATCCGCTCCGAGATG
0559	5′-flank to *malE* with an overhang matching the P*_gapDH_*-*cat*-*hpt* cassette	tccgggaaaacaaatcaaccTTGATTCTACCTCCAAATTA
0560	5′-flank to *malE* with an overhang matching the pDGO145 backbone	acattaacctataaaaataggcgtatcacgaggcgatatcCTGTGGGAAAAGTTATAGGT
0561	3′-flank to *malE* with an overhang matching the P*_gapDH_*-*cat*-*hpt* cassette	cctcgggcaaaaaaatcttttccatgcctattcccacgatAAGCCCAATATAATTACTGTCGC
0562	3′-flank to *malE* with an overhang matching the *pyk* gene from *T. saccharolyticum*	gtatagtgaatataaaatagCCGGAGTTGCAAGAATATAAAAC
0563	*pyk* gene from *T. saccharolyticum* with an overhang matching the 3′-flank of *malE*	ttatattcttgcaactccggCTATTTTATATTCACTATACCTTTGTAGACCA
0552	*pyk* gene from *T. saccharolyticum* with an overhang matching the P*_eno_* promoter	atatgaagggagaatggagaATGCGTAGAACTAAGATAATATGCACG
0553	P_eno_ promoter with an overhang matching *pyk*	attatcttagttctacgcatTCTCCATTCTCCCTTCATATAGC
0564	P_eno_ promoter with an overhang matching the 5′-flank of malE	taatttggaggtagaatcaaGGTTGATTTGTTTTCCCGGAA
1001	5′-flank to gogat with an overhang matching the pDGO145 backbone	attttgtttcccataggcgcgccgatTCCCCCCTCATCATGGGGAATATATGTGGG
1002	5′-flank to gogat with an overhang matching its 3′-flank	tattttgcagaatacatgaggcggccgcTAGCCGAGAATATGGCCAAAGGAGGT
1003	3′-flank to gogat with an overhang matching its 5′-flank	tagcggccgcCTCATGTATTCTGCAAAATACATTTTTT
1004	3′-flank to gogat with an overhang matching the PgapDH-cat-hpt cassette	attaattttttaaATACTTTCGCTTCTCCTCTATGAGCT
1005	PgapDH-cat-hpt cassette with an overhang matching the 3′-flank of gogat	agaggagaagcgaaagtatTTAAAAAATTAATTATTTTTTATCTAAACTATTGAA
1006	PgapDH-cat-hpt cassette with an overhang matching the int region of gogat	tccggaggatgtcggagagttTTATGAATACATTTCAGGTTTCAAAACGCC
1007	int region of gogat with an overhang matching the PgapDH-cat-hpt	aatgtattcataaAACTCTCCGACATCCTCCGGAAAACC
1008	int region of gogat with an overhang matching the pDGO145 backbone	tatacactccgctagcgcggatccgattTGAAAGAAGGTCAAATAAGGATACCGTCGGG
0033	Sequencing of *hpt* region in pJY19; confirmation of P*_gapDH_*-*cat*-*hpt* selection cassette removal	GCTATCTTTACAGGTACATCATTCTGTTTGTG
0034	Sequencing of *hpt* region in pJY19; confirmation of P*_gapDH_*-*cat*-*hpt* selection cassette removal	TTTCATCAAAGTCCAATCCATAACCC
0050	Sequencing of int region in pJY19	CCAGACGAAAAAGTTTTGAC
0051	Sequencing of int region in pJY19	TATGTCACGCTTACATTCAC
0284	Sequencing of int to *hpt* region in pJY19	GTTAGAGCGGCATTATCCCT
0555	Sequencing of backbone to 5′-flank in pJY19	GAGCGGATACATATTTGAATG
0556	Sequencing of 5′-flank to P*_eno_*-*pyk* region in pJY19	AGTTGGTATGAATTTATTCC
0565	Sequencing of 5′-flank to backbone in pJY19	TCTATTCCATACATGCCG
0566	Sequencing of 3′-flank to P*_gapDH_* region in pJY19	GCATATAAGATCCGCTTTC
0567	Sequencing of 3′-flank to *pyk* region in pJY19 and AVM064	GAAGCCGTAAGTGTGGATG
0568	Sequencing of 5′-flank to pyk in AVM064	ATCCATCGTAAATTCCGAAG
0281	Sequencing of P*_gapDH_* to 3′-flank region in pJY19	AAGAAAACAGACGCGCCC
0283	Sequencing of *pyk* to 3′-flank region in pJY19 and AVM064	ATCATCGTTGCTCAAAAAACTG
0159	Sequencing of *tdk* region in pJY19	GGTACGAATGTATAAGATGGTGC
0267	Sequencing of *tdk* region in pJY19	TTCTCCTGCCACATGAAG
0286	Sequencing of *tdk* region in pJY19	GCATGTTGTCGCCGTTATGA
0232	Sequencing of *pyk* region in pJY19	ATGCGTAGAACTAAGATAATATGCACG
0275	Sequencing of *pyk* region in pJY19 and AVM064	GAGATATAGGCGCAACTGCC
0276	Sequencing of *pyk* region in pJY19 and AVM064	GCGACAGGTATTCCTGCCGA
0277	Sequencing of *pyk* region in pJY19 and AVM064; confirmation of P_eno_-*pyk* insertion in AVM064	GGAATCGACATGATTGCAGCG
0278	Sequencing of *pyk* region in pJY19 and AVM064	TTTCGACACCCAAATCGCCG
0279	Sequencing of *pyk* to P*_eno_*-5′-flank in pJY19 and AVM064	CAGGCCCTTTTGTATCAAGC
1009	Sequencing of gogat region in pNJ22::GOGAT_del	ACGCACGAAAAGCCCTCTAG
1010	Sequencing of gogat region in pNJ22::GOGAT_del	AGGCGAGTCGGATAAATTTC
1011	Sequencing of gogat region in pNJ22::GOGAT_del	AATAAAAACGGTGAGTTTCC
1012	Sequencing of gogat region in pNJ22::GOGAT_del	GACGGAGAGTTAGGTTATTGGG
1013	Sequencing of gogat region in pNJ22::GOGAT_del	ACAGAAGAAGAGTTGAAGGAAAAG
1014	Sequencing of gogat region in pNJ22::GOGAT_del	CATCCTCCGGAAAACCTACC
0285	Confirmation of P*_cbp_*-*tdk* selection marker removal	ACGTTATATTGCTTGCCGGG
0289	Confirmation of P*_cbp_*-*tdk* selection marker removal	AAGACTCCTTTGCTCCAACC
0569	Confirmation of *malE* deletion, P_eno_-*pyk* insertion	CTTGTAGTATCCAATCCTGTTGACA
0472	Confirmation of *malE* deletion, P_eno_-*pyk* insertion	GTTGTAATGGTAAGCTGTTGCG
0544	Confirmation of *malE* deletion	GAGACATAGGACCTGAAGCC
0570	Confirmation of *malE* deletion	GCTCTTACATCAAGCGCACC
0238	Confirmation of *hpt* deletion	CACTTTCTTGTTGGCTCTGGCAGC
0239	Confirmation of *hpt* deletion	CCGGAGATGAGGCTTTTGTTGAGAAC
0003	Confirmation of *nfnAB* deletion	TCATCCACCCACGGTACT
0004	Confirmation of *nfnAB* deletion	GGGGGAAATGTATAAGAGGGGA
0681	Confirmation of *gogat* deletion	AGAACATATTAAAACCCCGGCA
0682	Confirmation of *gogat* deletion	TGGGTAAAATGGGTTGCTCC
1015	Confirmation of *gogat* deletion	CTATCTTTTTGTTTATCTTCATTACACATC
1016	Confirmation of *gogat* deletion	GATTTGCAGGTTATGGAATCTATC
1017	Confirmation of *gogat* deletion	CAGACAATTTTCTATGACAATATCTTTATC
1018	Confirmation of *gogat* deletion	TACAGGTTTGATAAAAGATATAAACGG
1019	Confirmation of *gogat* deletion	TCTATTTCATGTTGCCCGGG
1020	Confirmation of *gogat* deletion	AACCATACTTCCGAATACATTGGG

a The annealing sequences are represented by uppercase letters. The overhang sequences used for the Gibson assembly are represented by lowercase letters.

### Media and culture conditions.

Serum bottle cultivations were carried out in 125-mL Wheaton serum bottles (DWK Life Sciences, Millville, NJ, USA) that were closed with blue butyl rubber stoppers (number CLS-4209-14, Chemglass Life Sciences, NJ, US) and contained 50 mL of a defined low-carbon (LC) medium (56, 58). Per L, the medium contained 5 g D-(+)-cellobiose, 0.5 g urea, 5 g MOPS, 2 g KH_2_PO_4_, 3 g K_2_HPO_4_, 0.1 g Na_2_SO_4_, 0.2 g MgCl_2_·6H_2_O, 0.05 g CaCl_2_·2H_2_O, 0.0035 g FeSO_4_·7H_2_O, 0.025 g FeCl_2_·4H_2_O, 0.1 g l-cysteine-HCl-monohydrate, vitamins (0.02 g pyrodoxamine dihydrochloride, 0.004 g p-aminobenzoic acid, 0.002 g d-biotin, and 0.002 g vitamin B_12_), and trace elements (1.25 mg MnCl_2_·4H_2_O, 0.5 mg ZnCl_2_, 0.125 mg CoCl_2_·6H_2_O, 0.125 mg NiCl_2_·6H_2_O, 0.125 mg CuSO_4_·5H_2_O, 0.125 mg H_3_BO_3_, and 0.125 mg Na_2_MoO_4_·2H_2_O). The suppliers of the chemicals are listed in Yayo et al. (56). The final medium for the closed-batch serum bottles was prepared from sterile anaerobic stock solutions as described by Kuil et al. (30) and purged with 20% CO_2_ and 80% N_2_ (Strandmöllen AB) prior to inoculation by alternating between vacuum and gas for five cycles.

For chemostats, the LC medium described above was used, except with 1 g L^−1^ cysteine, no MOPS, and 5 times higher trace element concentrations, as described by Yayo et al. (56), and the nitrogen source was changed from urea to ammonium to avoid the cyclic CO_2_ production observed in preliminary carbon-limited chemostats with urea (at a dilution rate of 0.1 h^−1^). With urea in the feed, a spike in the off-gas CO_2_ concentration every 30 h was found to be coupled to the alternating breakdown and accumulation of urea and ammonium (data not shown). Instead, the chemostat feed contained 0.25 g L^−1^ or 2 g L^−1^ ammonium chloride (equal to 0.07 or 0.9 g L^−1^ ammonium) (Sigma) for the ammonium-limited or cellobiose-limited cultivations, respectively (15). The final medium (10 L) was prepared in 10 L Duran flasks (Saveen and Werner AB, Malmö, Sweden) from stock solutions as described by Yayo et al. (56). The feed vessels were covered in aluminum foil and were continuously stirred and sparged with filtered N_2_ gas (99.999%) (Nippon Gases, Köping, SE, or Air Liquide Gas AB, Malmö, Sweden) at room temperature.

The inocula for serum bottle cultivations and chemostats were prepared by two transfers from the freezer stock in serum bottles. First, an overnight culture was inoculated into 50 mL of fresh medium. At an OD of 0.5 to 1.0, this preculture was transferred to the main cultivation (serum bottle or bioreactor) with an inoculation volume of 5%. All of the serum bottles were incubated in a Jeio Tech ISS-4075R incubator (Milmedtek AB, Karlskrona, Sweden) at 55°C and 180 rpm.

### Chemostats.

Chemostats were performed in a multiparallel stainless-steel bioreactor system (GRETA from Belach AB, Stockholm, Sweden) with up to 6 unpressurized bioreactors running simultaneously at 55°C with 400 rpm agitation. The working volume was maintained at 0.8 L via feedback regulation on the effluent pump via a level sensor. The headspace of 0.45 L was continuously purged with filtered N_2_ gas (N5.0, Nippon Gases or Air Liquide Gas) at 0.2 L min^−1^, and the outgas was passed through a condenser. In order to minimize O_2_ permeability, Viton O-rings, Viton septa, and black Tygon tubing (A-60-G) were used (Sigma-Aldrich). The pH was measured with a SteamLine pH electrode (SL80-225pH from VWR) and was controlled at 7.0 via the addition of filter-sterilized 4.0 M KOH (0.2 μm PES filters) (VWR). The base vessel and 10 L feed vessels were connected via sterile 0.6 mm needles through septa on the head-plate (one feed vessel per reactor). A feed rate of 0.08 L per min, corresponding to a dilution rate of 0.10 h^−1^, was set and resulted in minimal intervals between medium droplets, thereby avoiding feast-famine cycles. The flow rate was checked daily with in line glass serological pipettes (25 mL) that were connected to the feed tube via a T-connection. Effluent was collected from a steam-sterilized sample valve in the bottom of the bioreactor into sterilized plastic Nalgene bottles (VWR) equipped with gas exhaust filters. The septa were cleaned with 70% isopropanol before and after the inoculation, sampling, and addition of feed and base tubes.

A batch cultivation preceded the chemostat phase in the bioreactors and was monitored via base titration or offline optical density measurements (see below). The feed pump, effluent pump, and level sensor were started at the end of the batch or shortly thereafter.

Steady-state was defined as a change of less than 5% in the biomass concentration over 3 residence times and after at least 4 residence times from the feed start. Pre-steady-state samples (35 mL) were taken in order to measure the OD and cell dry weight (CDW, see below) and thereby determine whether steady-state had been reached. After this, steady-state samples (described below) were taken (9 to 12 residence times after the feed start).

To investigate chemostat homogeneity, the OD and CDW of both the reactor and the effluent were compared. No significant differences (>5%) were observed for any of the characterized strains or conditions.

The culture purity of the steady-state cultures was assessed via the microscopy and sequencing of the 16S rRNA locus, using primers 0001 and 0002 (Table 7). Cross-contamination was excluded via the targeted PCR amplification of the strain-specific genotypes.

### H_2_ analysis.

The headspace H_2_ mole fraction was measured via offline mass spectrometry. Samples were collected for each bioreactor in serum bottles that were closed with thick butyl rubber stoppers (number CLS-4209-14, Chemglass Life Sciences) to prevent gas diffusion (only for strains LL345 and LL1715). Gas was flowing through the bottles continuously from the start of the cultivations. These bottles were connected to the tops of the condensers via septa, sterile 0.6 mm needles, 0.2 μm syringe filters (PES), and Norprene tubing (3 mm ID). The bottles were immediately detached prior to the liquid sampling, and they were stored at room temperature until analysis on the same or next day.

The mass spectrometer consisted of an Extrel RGA mounted on an ultrahigh vacuum (<10^−7^ Pa) chamber. Sample gases were injected through a blue butyl rubber stopper (number CLS-4209-14, Chemglass Life Sciences) into a small, high-pressure region (pumped between samples), from where the gas was let into the UHV region with a precision leak valve. The pressure in the UHV chamber was monitored and controlled during the measurements, typically at 4.5 ± 0.8 · 10^−5^ Pa. 20 mass spectra were averaged to reduce the effects of any possible changes in pressure during each measurement. The mass spectra for the background subtraction were measured prior to each sample.

Ions were detected using a Faraday cup. The different ionization efficiencies, the fragmentation of the different species, and the *m/z*-dependent transmission efficiency of the quadrupole were accounted for via calibration with gas mixtures of known composition (0.2%, 0.5%, and 2% H_2_ in 20% O_2_ and balance N_2_; 1% H_2_ in 99% N_2_ [Skandinaviska Gasprodukter AB, Södertälje, Sweden]; 5% H_2_ in 10% CO_2_ and 85% N_2_ [Strandmöllen, Ljungby, Sweden]). This was done by determining calibration values for each of the parent ions that yielded the correct, known composition. This simple method was only valid for the species in the calibration mixtures but sufficed, as other species (e.g., ethanol) were not observed in the mass spectra of the samples.

### Extracellular metabolite analysis.

A culture sample of approximately 100 mL was quickly withdrawn from the reactor and stored on ice. Samples from the feed vessels were collected at the beginning and end of the cultivation. Aliquots of 1 mL were centrifuged in a tabletop centrifuge (Eppendorf 5424, Thermo Fisher Scientific, Stockholm, Sweden) at 20,238 × *g* for 2 min. The supernatant was transferred to a nylon, low protein-binding, 0.22 μm Corning Costar Spin-X centrifuge tube filter (Sigma-Aldrich) and centrifuged at 20,238 × *g* for 2 min. Part of the filtrate was stored at 4°C until the analysis of the sugars and canonical fermentation products on an HPLC system, whereas the rest of the filtrate was stored at −20°C until the analysis of the ammonium, proteins, and amino acids (described below).

The supernatant samples stored at 4°C were analyzed within 1 week for cellobiose, glucose, ethanol, acetate, formate, lactate, pyruvate, and malate on a Waters Alliance 2695 HPLC system (Waters Sverige AB, Solna, Sweden) equipped with a Bio-Rad Aminex HPX-87H column (Bio-Rad, Solna, Sweden) that was operated at 60°C and with a 0.6 mL min^−1^ flow rate. The formate, pyruvate, and malate were separated using a 75 mM H_2_SO_4_ mobile phase, whereas the remaining components were separated using a 5 mM H_2_SO_4_ mobile phase. Cellobiose, glucose, and ethanol were detected using a Waters 2414 refractive-index detector, and the acids were detected using a Waters 2996 photodiode-array detector at 210 nm.

The amino acids were analyzed via derivatization, using a Waters AccQ-Tag Ultra commercial kit (cat. no. WAT052880) (Waters Sverige AB, Solna, Sweden). First, 2-aminobutyric acid (AABA) (Sigma-Aldrich) was added as an internal standard. Then, supernatant protein was removed via acidification with 5 g L^−1^ trichloroacetic acid (Supelco from Sigma-Aldrich), incubation for 20 min at room temperature, and centrifugation at 20,238 × *g* for 10 min in a tabletop centrifuge (Eppendorf 5424, Thermo Fisher Scientific, Stockholm, Sweden). The supernatant was derivatized according to the AccQ-Tag commercial kit protocol (Waters “UPLC Amino Acid Analysis Solution System Guide,” 71500129702, revision B). Separation was performed in a Waters ACQUITY Ultra-Performance Liquid Chromatography (UPLC) system with a reverse phase-UPLC AccQ-Tag Ultra column (cat.no. WAT052885) at 55°C with a flow rate of 0.70 mL min^−1^ and a run time of 10 min. The mobile phase consisted of eluent A, prepared by diluting Waters AccQ-Tag Ultra Eluent A concentrate (cat. no. 186003838) 20 times in ultrapure water (Purelab Chorus from AB Ninolab [Stockholm, Sweden]), and eluent B, as provided by Waters (cat. no. 186003839). The following elution profile was used: initial 99.9% A, 0.1% B; 0.54 min 99.9% A, 0.1% B, curve 6; 3.80 min, 96.0% A, 4.0% B, curve 6; 4.20 min, 96.0% A, 4.0% D, curve 6; 5.74 min, 90.9% A, 9.1% B, curve 7; 7.74 min, 78.8% A, 21.2% B, curve 6; 8.04 min 40.4% A, 59.6% B, curve 6; 8.05 min, 10.0% A, 90.0% B, curve 6; 8.64 min, 10.0% A, 90.0% B, curve 6; 8.73 min, 99.9% A, 0.1% B, curve 6; and 9.5 min, 99.9% A, 0.1% B, curve 6. Tryptophan, cysteine, and tyrosine were quantified using an ACQUITY TUV detector at 250 nm. Alanine, arginine, asparagine, aspartate, glutamine, glycine, glutamate, histidine, isoleucine, leucine, methionine, phenylalanine, serine, threonine, and valine were detected using a ACQUITY FLR detector with excitation at 266 nm and emission at 473 nm. The UPLC was set-up according to the AccQ-Tag commercial kit protocol. As standards, a Waters amino acid standard solution with 17 amino acids (cat. no. WAT088122) was mixed with glutamine (Irvine Scientific, Tilburg, The Netherlands), asparagine, and tryptophan (Sigma-Aldrich). A coelution of arginine and glutamine was observed.

Extracellular ammonium in the culture supernatants and medium samples was measured spectrophotometrically, using a commercial MegaZyme Ammonium Assay Kit (cat. no. KURAMR, Megazyme, Bray, Ireland) and a standard curve with ammonium chloride (Sigma-Aldrich).

### Extracellular protein analysis.

The extracellular supernatant protein was quantified using a Bradford assay (59) that was modified for the detection of lower protein concentrations (1 to 10 μg mL^−1^). The protocol described in a Sigma-Aldrich technical bulletin (cat. no. B6916) was used with a 96-well microplate (flat-bottom, clear, PS) (Sigma-Aldrich) and with bovine serum albumin (Sigma-Aldrich) as the standard. A conversion to molar carbon, nitrogen, and degree-of-reduction was done using an estimated general amino acid composition for *C. thermocellum*, which was derived by counting the occurrences of each amino acid in all of the open-reading frames of the genome and correcting for polymerization (loss of H_2_O) (Table S1). These elemental concentrations were used to calculate the respective recovery on the substrate (described below) (Tables S3–S5).

### CDW and OD measurements.

The cell dry weight was quantified in technical triplicates by first transferring 10 mL of culture into dried, preweighed, conical, glass centrifuge tubes and centrifuging at 2,250 × *g* in a tabletop centrifuge (Z206 A, Hermle Labortechnik GmbH, Wehingen, Germany) for 20 min. After washing the pellet in an equal volume of ultrapure water (Purelab Chorus), another round of centrifugation was performed. The washed pellet was dried overnight in a VENTI-Line forced-convection oven (VWR) set at 105°C. Upon cooling for 1 h in a desiccator, the dried glass tube was weighed. The CDW was calculated by dividing the difference in tube weights with the sample volume. The optical density was measured in technical triplicates in 1 mL polystyrene cuvettes in a V-1200 spectrophotometer (VWR) at 600 nm. Deionized water was used as a blank and for the dilutions.

### Glycogen determination.

The sampling and assay of the glycogen content was performed as previously described (30). Briefly, 1 mL of sample was mixed with 5 mL of ice-cold methanol (–80°C) and centrifuged (10,000 × *g*, 10 min, −10°C). The pellet was washed with 5 mL of methanol (–80°C) and centrifuged again. After removing the supernatant, the pellet was stored at −80°C until analysis. Glycogen was hydrolyzed to glucose and was analyzed on a HPLC system (as described above).

### Calculation of yields and rates.

The quantification of the extracellular metabolites and the biomass concentration in the chemostats allowed for the calculation of the yields on the substrate (Y_i/S_) and the biomass-specific rates (q) at steady-state. A general mass balance for a metabolite i in the liquid phase is described by Equation 1.

(1) $$\frac{d\left(C_{i}\text{ *}V_{L}\right)}{\mathrm{dt}}=F_{\mathrm{in}}\text{ *}C_{i,\mathrm{in}}-F_{\mathrm{out}}\text{ *}C_{i,\mathrm{out}}\pm q_{i}\text{ *}C_{x}\text{*}V_{L}\pm T_{N,i}$$

Here, C denotes concentrations (mmol L^−1^) for metabolite i or biomass x, $V_{L}$ is the liquid volume (L), F is the flow rate (L h^−1^), $q_{i}$ is the biomass-specific conversion rate (mmol g_x_^−1^ h^−1^), and $T_{N,i}$ is the transfer rate between the gas and liquid phases for volatile compounds (mmol g_x_^−1^ h^−1^). Both $q_{i}$ and $T_{N,i}$ are defined as positive numbers, and the sign in front of each term depends on the production/transfer into liquid (+) or consumption/transfer out of liquid (−). Assuming steady-state $\left(\frac{d\left(C_{i}*V_{L}\right)}{\mathrm{dt}}=0\right)$, ideal mixing ($C_{i,\mathrm{out}}=C_{i}$), a constant volume, $F=F_{\mathrm{in}}=F_{\mathrm{out}}$, and the definition of the dilution rate as $D=\frac{F}{V}$ (h^−1^), an expression for $q_{i}$ can be derived.

(2) $$q_{i}=\pm \frac{D\left(C_{i,\mathrm{in}}-C_{i}\right)\pm T_{N,i}}{C_{x}}$$

With a condenser to minimize evaporation, the transfer rate, $T_{N,i}$, was assumed to be zero for all of the aqueous metabolites, except ethanol. For ethanol, a first-order transfer rate coefficient was determined to be $k_{\mathrm{ethanol}}$ = 0.018 ± 0.01 h^−1^ (*n *= 3) in the specified bioreactor system at 55°C and 400 rpm with 0.2 L min^−1^ overhead purging with N_2_ gas. This determination was made by measuring the dissipating ethanol concentrations in the liquid phase over time (data not shown). Therefore, the transfer term in Equation 2 was $-k_{\mathrm{ethanol}}*C_{\mathrm{ethanol}}*V_{L}$, giving the following expression for the ethanol production rate (where $C_{\mathrm{ethanol},\mathrm{in}}=0$).

(3) $$q_{\mathrm{ethanol}}=\frac{\left(D+k_{\mathrm{ethanol}}\right)\text{*}C_{\mathrm{ethanol}}}{C_{x}}$$

Based on the biomass-specific conversion rates, the yield of product i on the consumed substrate cellobiose S was calculated as follows.

(4) $$Y_{\mathrm{iS}}=\frac{q_{i}}{q_{S}}$$

In order to calculate the molar yield of biomass on cellobiose, a molecular weight of 24.66 g Cmol^−1^ and a degree-of-reduction of 4.30 were used, based on the biomass composition of CH_1.71_O_0.43_N_0.20_S_0.01_ with 4.32% ash content from Hogsett (60) and the following elemental degree-of-reductions: C = 4, H = 1, O = −2, N = −3, S = 6.

The estimated rate of CO_2_ production was calculated based on the current biochemical knowledge regarding the *C. thermocellum* metabolism and the genome-scale metabolic model iCBI655 (16). In addition, in the conversion of cellobiose into biomass, CO_2_ is produced as a byproduct to balance the carbon, as biomass is slightly more reduced per carbon than is cellobiose. Based on the carbon and degree-of-reduction balancing of that biosynthesis reaction, it was estimated that 3.04 mmol CO_2_ per g biomass is formed. With this, the total CO_2_ production rate was calculated based on measured conversion rates, as shown in Equation 5 below.

(5) $$q_{\mathrm{CO}2}^{\mathrm{total}}=q_{\mathrm{acetate}}+q_{\mathrm{ethanol}}-q_{\mathrm{formate}}-q_{\mathrm{arginine}}+2{\mathrm{* q}}_{\mathrm{isoleucine}}+2*q_{\mathrm{leucine}}+q_{\mathrm{lysine}}+q_{\mathrm{phenylalanine}}+q_{\mathrm{tryptophan}}+q_{\mathrm{tyrosine}}+q_{\mathrm{valine}}-q_{\mathrm{malate}}+3.04\text{ *}\mu$$

The H_2_ production rate was derived by combining the mass balances for the gas and liquid phases. First, the general mass balance in the gas phase, as shown in Equation 6, can be simplified for H_2_, as the inlet gas only consists of N_2_.

(6) $$\frac{d\left(y_{H_{2}}\text{ *}N_{g}\right)}{\mathrm{dt}}=F_{N,\mathrm{in}}\text{ *}y_{H_{2},\mathrm{in}}-F_{N,\mathrm{out}}\text{ *}y_{H_{2},\mathrm{out}}+T_{N,H_{2}}$$

At steady-state, Equation 7 is valid.

(7) $$F_{N,\mathrm{out}}\text{ *}y_{H_{2},\mathrm{out}}=T_{N,H_{2}}$$

Here, $y_{i}$ denotes the mole fraction of the gas, $N_{g}$ is the total gas amount (mol), and $F_{N}$ is the gas flow in or out of the bioreactor (mol h^−1^). The H_2_ production rate was derived by combining Equation 1 for H_2_ (simplified by neglecting the inflow and outflow of H_2_ in the liquid due to low solubility) with Equation 7.

(8) $$q_{H2}=\frac{F_{N,\mathrm{out}}*y_{H_{2},\mathrm{out}}}{C_{x}\text{ *}V_{L}}$$

Here, $F_{N,\mathrm{out}}$ (mol h^−1^) could be estimated from a nitrogen balance on the inlet (pure N_2_) and outlet (N_2_, CO_2_, and H_2_) gas streams (N_2_ is inert) and the use of the ideal gas law.

### Calculation of the carbon, nitrogen, and degree-of-reduction recoveries on the consumed substrate.

The recovery on the substrate was defined as the ratio between the products and the substrates, in regard to their carbon, nitrogen, or degree-of-reduction contents. The substrate for the carbon and degree-of-reduction calculations was cellobiose, and the substrate for the nitrogen calculations was ammonium. In the degree-of-reduction balancing, the following definitions were made for the elements: C = 4, H = 1, O = −2, N = −3, S = 6. The molar concentrations of biomass and protein were estimated based on the molecular weights specified above.

### Enzyme activity assays.

The cell-free extracts were prepared as described previously (56). In the case of steady-state cultures, 50 mL of sample was harvested. Enzyme activities were assayed aerobically at 55°C in a Cary 50 UV-visible spectrophotometer with a single-cell Peltier element from Varian AB (Solna, Sweden). The conversion of NAD(P)^+^ to NAD(P)H was followed at 340 nm in quartz cuvettes (VWR) with a path length of 1 cm and a reaction volume of 1 mL. An extinction coefficient of 6.22 AU mmol^−1^ L cm^−1^ was used for NAD(P)H. The proportionality between the activity and the amount of cell extract was confirmed by assaying two different concentrations of cell extract in technical duplicates. Each activity is reported for biological duplicates. An assay was carried out by first incubating all reaction mixture components, except for the coupling enzymes and the cell extract, at 55°C for 5 min. Then, the coupling enzymes (if used) and the cell extract were added and equilibrated for an additional 4 min, with the last minute serving as a background slope. The reaction was initiated by adding the indicated metabolites. The slope for the first 30 s was used to calculate the activity.

The glutamate dehydrogenase activity was measured based on the oxidation rate of NAD(P)H as 2-ketoglutarate was converted to glutamate (15, 61). The assay mixture contained 50 mM Tris-HCl (pH 8.0 at 55°C), 5 mM DTT, 50 mM NH_4_Cl, 5 mM 2-ketoglutarate (pH 8.0 at 25°C), and 0.3 mM NADPH or NADH. The reaction was started via the addition of 2-ketoglutarate.

The malic enzyme activity was measured by following the reduction of NADP^+^ as malate was converted to pyruvate (32). The assay mixture contained 50 mM Tris-HCl (pH 7.5 at 55°C), 5 mM DTT, 2 mM NADP^+^, 20 mM NH_4_Cl, 2.5 mM malate, and 50 or 100 μL cell extract. The reaction was started via the addition of malate.

The pyruvate phosphate dikinase activity was assayed by coupling the formation of pyruvate from PEP to the formation of lactate from pyruvate via added lactate dehydrogenase, resulting in the oxidation of NADH (32). The assay mixture contained 50 mM Tris-HCl (pH 7.5 at 55°C), 5 mM DTT, 0.3 mM NADH, 5 mM MgCl_2_·6H_2_O, 20 mM NH_4_Cl, 2 mM PEP, 2 mM AMP, 13 U mL^−1^ l-lactate dehydrogenase (from bovine heart, Sigma L2625), 1 mM K_4_PP_i_, and 50 or 100 μL cell extract. The reaction was started via the addition of PP_i_.

The pyruvate kinase activity was measured by coupling the formation of pyruvate from PEP to the formation of lactate from pyruvate via added lactate dehydrogenase, resulting in the oxidation of NADH (32). The assay mixture contained 50 mM Tris-HCl (pH 7.5 at 55°C), 5 mM DTT, 0.3 mM NADH, 12 mM MgCl_2_·6H_2_O, 10 mM KCl, 10 mM ADP, 0.1 mM 3-phosphoglyceric acid, 5 mM PEP, 13 U mL^−1^ l-lactate dehydrogenase (from bovine heart, Sigma L2625), and 50 or 100 μL cell extract. The reaction was initiated via the addition of PEP.

The lactate dehydrogenase (LDH) activity was assayed by measuring the oxidation of NADH as pyruvate was converted to lactate (62). The mixture contained 10 mM pyruvate, 1 mM fructose 1,6-bisphosphate, 0.22 mM NADH, 200 mM Tris-HCl (pH 7.3 at 55°C), and 50 or 100 mL of cell extract. The reaction was initiated via the addition of pyruvate. LDH activity was used routinely as a quality control on all cell extracts (Table S8).

### Data analysis.

Student’s *t* tests were used for the unpaired comparisons between values in this study.

### Data availability.

The data on the measured concentrations from the chemostats that were used in the calculations of the yields and rates are presented in Table S7. The GenBank accession numbers for the plasmids generated in this study are presented in Table 6.
