# Supplementary material for: The Roles of Nicotinamide Adenine Dinucleotide Phosphate Reoxidation and Ammonium Assimilation in the Secretion of Amino Acids as Byproducts of Clostridium thermocellum
Source: Appl Environ Microbiol. 2023 Jan 10;89(1):e01753-22. doi: 10.1128/aem.01753-22 (PMC9888227; doi:10.1128/aem.01753-22)
Supplement: Supplemental file 1 — Tables S1 to S8. Download aem.01753-22-s0001.pdf, PDF file, 0.4 MB [file aem.01753-22-s0001.pdf]

SUPPLEMENTAL TABLES

TABLE S1. Molecular weight and elemental composition of a general protein in the *C. thermocellum* ATCC 27405 genome by counting the occurrences of each amino acid in all open-reading frames and correcting for polymerization (loss of H_2O).

Amino acid	Molecular formula[a]	$M_{W,amino\ acid}$ (g/mol)	$M_{W,amino\ acid}$ corrected for polymerization (g/mol)	Count (all ORFs)	% Prevalance (mol_{aa}/mol_{prot})	$M_{W,protein}$ (g_{aa}/mol_{prot})	Element in protein (mol/mol_{prot}) C	H	N	O	S
Alanine	$C_3H_7NO_2$	89.1	71.1	68886	6.38	4.53	0.19	0.45	0.06	0.13	0.00
Arginine	$C_6H_{15}N_4O_2^+$	175.2	157.2	47155	4.37	6.87	0.26	0.66	0.17	0.09	0.00
Asparagine	$C_4H_8N_2O_3$	132.1	114.1	58251	5.39	6.16	0.22	0.43	0.11	0.16	0.00
Aspartate	$C_4H6NO_4^-$	132.1	114.1	61489	5.69	6.50	0.23	0.34	0.06	0.23	0.00
Cysteine	$C_3H_7NO_2S$	121.2	103.1	12654	1.17	1.21	0.04	0.08	0.01	0.02	0.01
Glutamate	$C_5H_8NO_4^-$	146.1	128.1	82288	7.62	9.76	0.38	0.61	0.08	0.30	0.00
Glutamine	$C_5H_{10}N_2O_3$	146.2	128.1	27732	2.57	3.29	0.13	0.26	0.05	0.08	0.00
Glycine	$C_2H_5NO_2$	75.1	57.1	72154	6.68	3.81	0.13	0.33	0.07	0.13	0.00
Histidine	$C_6H_9N_3O_2$	155.2	137.1	15430	1.43	1.96	0.09	0.13	0.04	0.03	0.00
Isoleucine	$C_6H_{13}NO_2$	131.2	113.2	91626	8.49	9.60	0.51	1.10	0.08	0.17	0.00
Leucine	$C_6H_{13}NO_2$	131.2	113.2	93461	8.66	9.79	0.52	1.13	0.09	0.17	0.00
Lysine	$C_6H_{15}N_2O_2^+$	147.2	129.2	87426	8.10	10.46	0.49	1.21	0.16	0.16	0.00
Methionine	$C_5H_{11}NO_2S$	149.2	131.2	27111	2.51	3.29	0.13	0.28	0.03	0.05	0.03
Phenylalanine	$C_9H_{11}NO_2$	165.2	147.2	45765	4.24	6.24	0.38	0.47	0.04	0.08	0.00
Proline	$C_5H_9NO_2$	115.1	97.1	36619	3.39	3.29	0.17	0.31	0.03	0.07	0.00
Serine	$C_3H_7NO_3$	105.1	87.1	65270	6.04	5.26	0.18	0.42	0.06	0.18	0.00
Threonine	$C_4H_9NO_3$	119.1	101.1	54463	5.04	5.10	0.20	0.45	0.05	0.15	0.00
Tryptophan	$C_{11}H_{12}N_2O_2$	204.2	186.2	9570	0.89	1.65	0.10	0.11	0.02	0.02	0.00
Tyrosine	$C_9H_{11}NO_3$	181.2	163.2	45643	4.23	6.90	0.38	0.46	0.04	0.13	0.00
Valine	$C_5H_{11}NO_2$	117.1	99.1	76806	7.11	7.05	0.36	0.78	0.07	0.14	0.00
Total		-	-	1079799	100	**112.73**	5.07	10.01	1.33	2.50	0.04
						Corrected for polymerization:	**5.07**	**8.01**	**1.33**	**1.50**	**0.04**

[a] Based on a pH of 7.0.

TABLE S2. Amino acid yields (mmol $mol_{cellobiose}^{-1}$) in chemostats with either cellobiose or ammonium as the sole limiting nutrient at a dilution rate of 0.10 h^{-1}.[a]

	**Cellobiose-limitation**					**Ammonium-limitation**				
	**Reference strain (LL345)**	**AVM064 (Δ*ppdk* Δ*malE*::P*eno*-*pyk*)**	**AVM003 (Δ*ppdk*)**	**LL1084 (Δ*nfnAB*)**	**AG1715 (Δ*gogat*)**	**Reference strain (LL345)**	**AVM064 (Δ*ppdk* Δ*malE*::P*eno*-*pyk*)**	**AVM003 (Δ*ppdk*)**	**LL1084 (Δ*nfnAB*)**	**AG1715 (Δ*gogat*)**
**Alanine**	6.5 ± 0.2	6.7 ± 0.4	6.7 ± 0.5	6.8 ± 0.2	5.6 ± 0.1	9.5 ± 0.1	6.1 ± 0.4	9.0 ± 0.4	8.0 ± 0.4	5.3 ± 0.4
**Asparagine**	<0.5	<0.5	<0.5	<0.5	<0.5	<0.5	<0.5	<0.5	<0.5	<0.5
**Aspartate**	<0.5	2.2 ± 0.3	1.0 ± 0.1	0.9 ± 0.5	1.1 ± 0.7	<0.5	0.8 ± 0.2	<0.5	0.7 ± 0.1	<0.5
**Glycine**	2.0 ± 0.2	2.4 ± 0.2	2.2 ± 0.2	1.9 ± 0.0	1.5 ± 0.2	2.2 ± 0.1	1.6 ± 0.2	1.9 ± 0.2	2.0 ± 0.2	<0.5
**Glutamate**	2.8 ± 0.1	2.7 ± 0.4	2.2 ± 0.1	3.1 ± 0.2	2.8 ± 0.2	5.8 ± 0.1	4.2 ± 0.2	4.9 ± 0.2	8.3 ± 0.4	5.5 ± 0.5
**Glutamine[b]**	<0.7 ± 0.0	<2.0 ± 0.4	<2.1 ± 0.7	<1.7 ± 0.2	<0.6 ± 0.1	<0.5	<2.1 ± 0.2	<2.8 ± 0.3	<1.0 ± 0.1	<0.5
**Histidine**	<0.5	<0.5	<0.5	<0.5	<0.5	<0.5	<0.5	<0.5	<0.5	<0.5
**Isoleucine**	1.3 ± 0.0	0.7 ± 0.0	1.5 ± 0.1	1.6 ± 0.2	1.1 ± 0.0	10.0 ± 0.2	3.7 ± 0.4	11.2 ± 2.4	9.1 ± 0.8	2.5 ± 0.8
**Leucine**	0.8 ± 0.0	1.4 ± 0.1	<0.5	0.7 ± 0.6	0.8 ± 0.0	2.5 ± 0.1	2.3 ± 0.2	2.9 ± 0.1	2.8 ± 0.1	1.1 ± 0.2
**Methionine**	<0.5	<0.5	<0.5	<0.5	<0.5	6.3 ± 0.5	<0.5	<0.5	0.9 ± 0.3	<0.5
**Phenylalanine**	1.1 ± 0.1	4.2 ± 0.6	1.2 ± 0.3	0.9 ± 0.2	0.9 ± 0.1	4.2 ± 0.1	4.4 ± 1.3	4.2 ± 0.5	5.8 ± 0.2	4.7 ± 1.0
**Serine**	2.6 ± 0.2	2.1 ± 0.1	2.3 ± 0.0	2.5 ± 0.1	2.0 ± 0.1	1.9 ± 0.2	1.4 ± 0.1	2.0 ± 0.1	2.0 ± 0.2	1.2 ± 0.5
**Threonine**	3.4 ± 0.3	3.8 ± 0.4	3.5 ± 0.4	3.6 ± 0.2	2.9 ± 0.3	3.6 ± 0.1	3.6 ± 0.4	3.0 ± 0.4	5.2 ± 0.5	<0.5
**Tryptophan**	<0.5	<0.5	<0.5	<0.5	<0.5	<0.5	<0.5	<0.5	<0.5	<0.5
**Tyrosine**	0.9 ± 0.0	0.8 ± 0.1	<0.5	<0.5	<0.5	1.9 ± 0.1	1.1 ± 0.1	1.4 ± 0.1	4.0 ± 1.0	<0.5
**Valine**	3.3 ± 0.1	1.3 ± 0.1	5.1 ± 0.3	5.8 ± 0.3	2.3 ± 0.1	67.3 ± 1.7	12.6 ± 2.8	62.3 ± 23.1	65.3 ± 8.3	16.0 ± 9.4

[a] Data are shown as average ± standard deviation for three biological replicates
[b] Estimated upper limit (see Materials and Methods)

TABLE S3. Carbon recovery on consumed cellobiose in chemostats limited on either cellobiose or ammonium at a dilution rate of 0.10 h^{-1}.[a]

% (Cmol/Cmol)	Cellobiose-limitation					Ammonium-limitation				
	Reference strain (LL345)	AVM064 (Δ*ppdk* Δ*malE*::P_{eno}-*pyk*)	AVM003 (Δ*ppdk*)	LL1084 (Δ*nfnAB*)	AG1715 (Δ*gogat*)	Reference strain (LL345)	AVM064 (Δ*ppdk* Δ*malE*::P_{eno}-*pyk*)	AVM003 (Δ*ppdk*)	LL1084 (Δ*nfnAB*)	AG1715 (Δ*gogat*)
**Biomass**[b]	15.49 ± 0.27	14.34 ± 0.61	16.79 ± 0.76	15.07 ± 0.04	15.79 ± 0.20	13.48 ± 0.12	13.21 ± 0.51	14.60 ± 0.64	13.49 ± 0.62	16.03 ± 0.57
**Glucose**	0.16 ± 0.02	0.06 ± 0.02	0.07 ± 0.03	0.03 ± 0.00	0.08 ± 0.03	0.59 ± 0.07	1.02 ± 0.18	1.56 ± 0.20	1.39 ± 0.12	1.55 ± 0.38
**Ethanol**	16.16 ± 0.97	25.78 ± 0.17	16.00 ± 0.55	16.04 ± 0.27	13.99 ± 1.10	17.76 ± 0.46	25.86 ± 0.89	14.06 ± 0.82	16.73 ± 0.42	12.91 ± 0.56
**Acetate**	23.04 ± 0.22	10.81 ± 0.30	24.29 ± 0.72	23.01 ± 0.31	23.00 ± 0.25	15.60 ± 0.10	10.09 ± 0.17	19.57 ± 2.00	16.85 ± 0.22	14.00 ± 1.13
**Formate**	0.96 ± 0.31	1.87 ± 0.16	0.81 ± 0.10	2.56 ± 0.06	0.62 ± 0.06	1.23 ± 0.09	2.64 ± 0.27	1.52 ± 0.43	1.04 ± 0.23	2.48 ± 1.02
**Lactate**	0.35 ± 0.03	0.51 ± 0.07	0.43 ± 0.05	0.30 ± 0.05	0.40 ± 0.05	1.68 ± 0.14	1.02 ± 0.41	1.53 ± 0.51	1.41 ± 0.10	0.92 ± 0.45
**Pyruvate**	0.09 ± 0.01	0.06 ± 0.02	0.33 ± 0.21	0.21 ± 0.04	0.27 ± 0.02	2.25 ± 0.12	0.28 ± 0.05	0.57 ± 0.11	0.77 ± 0.10	0.58 ± 0.29
**Malate**	0.16 ± 0.16	3.64 ± 0.62	<0.01	<0.01	<0.01	<0.01	1.13 ± 0.11	<0.01	0.07 ± 0.04	<0.01
**CO_2**[c]	19.83 ± 0.16	16.68 ± 0.36	20.66 ± 0.48	18.19 ± 0.26	19.11 ± 0.42	17.26 ± 0.23	16.29 ± 0.07	17.18 ± 0.69	17.55 ± 0.18	12.36 ± 0.97
**H_2**	0	0	0	0	0	0	0	0	0	0
**Ex. protein**[d]	3.94 ± 0.11	2.36 ± 0.18	3.19 ± 0.18	4.08 ± 0.14	3.40 ± 0.08	2.00 ± 0.08	1.50 ± 0.16	2.29 ± 0.14	1.87 ± 0.06	1.86 ± 0.25
**Alanine**	0.16 ± 0.01	0.17 ± 0.01	0.17 ± 0.01	0.17 ± 0.01	0.14 ± 0.00	0.24 ± 0.00	0.15 ± 0.01	0.22 ± 0.01	0.20 ± 0.01	0.13 ± 0.01
**Asparagine**	<0.01	<0.01	<0.01	<0.01	<0.01	<0.01	<0.01	<0.01	<0.01	<0.01
**Aspartate**	0.01 ± 0.02	0.07 ± 0.01	0.03 ± 0.00	0.03 ± 0.02	0.04 ± 0.02	0.01 ± 0.00	0.03 ± 0.01	<0.01	0.02 ± 0.00	<0.01
**Glycine**	0.04 ± 0.01	0.04 ± 0.00	0.04 ± 0.00	0.03 ± 0.00	0.03 ± 0.00	0.04 ± 0.00	0.03 ± 0.00	0.03 ± 0.00	0.03 ± 0.00	<0.01
**Glutamate**	0.12 ± 0.01	0.11 ± 0.02	0.09 ± 0.01	0.13 ± 0.01	0.12 ± 0.01	0.24 ± 0.01	0.17 ± 0.01	0.20 ± 0.01	0.35 ± 0.01	0.23 ± 0.02
**Glutamine/arginine**[e]	0.03 ± 0.00	0.08 ± 0.02	0.09 ± 0.03	0.07 ± 0.01	0.02 ± 0.00	0.02 ± 0.01	0.09 ± 0.01	0.12 ± 0.01	0.04 ± 0.00	0.02 ± 0.00
**Histidine**	<0.01	<0.01	<0.01	<0.01	<0.01	<0.01	<0.01	0.02 ± 0.00	<0.01	<0.01
**Isoleucine**	0.06 ± 0.00	0.03 ± 0.00	0.07 ± 0.00	0.08 ± 0.01	0.05 ± 0.00	0.50 ± 0.01	0.18 ± 0.02	0.56 ± 0.12	0.45 ± 0.04	0.12 ± 0.04
**Leucine**	0.04 ± 0.00	0.07 ± 0.01	<0.01	0.03 ± 0.03	0.04 ± 0.00	0.13 ± 0.01	0.11 ± 0.01	0.15 ± 0.00	0.14 ± 0.01	0.06 ± 0.01
**Methionine**	<0.01	<0.01	<0.01	<0.01	<0.01	0.26 ± 0.02	<0.01	0.02 ± 0.02	0.04 ± 0.01	<0.01
**Phenylalanine**	0.08 ± 0.01	0.31 ± 0.04	0.09 ± 0.02	0.07 ± 0.01	0.07 ± 0.00	0.31 ± 0.01	0.33 ± 0.10	0.31 ± 0.04	0.43 ± 0.02	0.35 ± 0.08
**Serine**	0.06 ± 0.01	0.05 ± 0.00	0.06 ± 0.00	0.06 ± 0.00	0.05 ± 0.00	0.05 ± 0.01	0.03 ± 0.00	0.05 ± 0.00	0.05 ± 0.00	0.03 ± 0.01
**Threonine**	0.11 ± 0.01	0.13 ± 0.01	0.12 ± 0.01	0.12 ± 0.01	0.10 ± 0.01	0.12 ± 0.00	0.12 ± 0.01	0.10 ± 0.01	0.17 ± 0.02	0.02 ± 0.01
**Tryptophan**	<0.01	0.03 ± 0.00	<0.01	<0.01	<0.01	<0.01	<0.01	<0.01	<0.01	<0.01
**Tyrosine**	0.07 ± 0.00	0.06 ± 0.01	0.04 ± 0.00	<0.01	<0.01	0.14 ± 0.01	0.08 ± 0.01	0.10 ± 0.01	0.30 ± 0.08	<0.01
**Valine**	0.14 ± 0.01	0.05 ± 0.00	0.21 ± 0.01	0.24 ± 0.01	0.09 ± 0.00	2.80 ± 0.07	0.53 ± 0.12	2.59 ± 0.96	2.72 ± 0.35	0.67 ± 0.39
**Sum of all products**	**81 ± 1**	**77 ± 0**	**84 ± 2**	**81 ± 1**	**77 ± 1**	**77 ± 1**	**75 ± 1**	**77 ± 3**	**76 ± 1**	**64 ± 1**
**Sum of amino acids**	**0.93 ± 0.05**	**1.22 ± 0.05**	**1.01 ± 0.07**	**1.04 ± 0.06**	**0.75 ± 0.04**	**4.86 ± 0.07**	**1.88 ± 0.29**	**4.49 ± 1.11**	**4.96 ± 0.34**	**1.64 ± 0.42**

[a] Data are shown as average ± standard deviation for three biological replicates.
[b] Based on a molecular weight of 24.66 g mol^{-1} for the biomass composition $CH_{1.71}O_{0.43}N_{0.20}S_{0.01}$ with 4.32 % ash content (60).
[c] Calculated from fermentation product yields and biomass yield (see Materials and Methods).
[d] Based on a molecular weight of a general protein (see Supplemental Table S2 and Materials and Methods).
[e] Estimated lower value based on 4 carbons per mole.

TABLE S4. Degree-of-reduction recovery on consumed cellobiose in chemostats limited on either cellobiose or ammonium at 0.10 h^{-1}.[a,b]

% (DR/DR)	Cellobiose-limitation					Ammonium-limitation				
	**Reference strain (LL345)**	**AVM064 (Δ*ppdk* Δ*malE*::P_{eno}-*pyk*)**	**AVM003 (Δ*ppdk*)**	**LL1084 (Δ*nfnAB*)**	**AG1715 (Δ*gogat*)**	**Reference strain (LL345)**	**AVM064 (Δ*ppdk* Δ*malE*::P_{eno}-*pyk*)**	**AVM003 (Δ*ppdk*)**	**LL1084 (Δ*nfnAB*)**	**AG1715 (Δ*gogat*)**
**Biomass**[c]	16.64 ± 0.29	15.42 ± 0.66	18.05 ± 0.81	16.20 ± 0.05	16.97 ± 0.22	14.49 ± 0.13	14.20 ± 0.55	15.69 ± 0.69	14.51 ± 0.67	17.23 ± 0.61
**Glucose**	0.16 ± 0.02	0.06 ± 0.02	0.07 ± 0.03	0.03 ± 0.00	0.08 ± 0.03	0.59 ± 0.07	1.02 ± 0.18	1.56 ± 0.20	1.39 ± 0.12	1.55 ± 0.38
**Ethanol**	24.24 ± 1.45	38.68 ± 0.25	24.00 ± 0.82	24.05 ± 0.40	20.98 ± 1.65	26.64 ± 0.70	38.79 ± 1.34	21.08 ± 1.22	25.09 ± 0.62	19.37 ± 0.84
**Acetate**	23.04 ± 0.22	10.81 ± 0.30	24.29 ± 0.72	23.01 ± 0.31	23.00 ± 0.25	15.60 ± 0.10	10.09 ± 0.17	19.57 ± 2.00	16.85 ± 0.22	14.00 ± 1.13
**Formate**	0.48 ± 0.16	0.94 ± 0.08	0.41 ± 0.05	1.28 ± 0.03	0.31 ± 0.03	0.62 ± 0.05	1.32 ± 0.13	0.76 ± 0.21	0.52 ± 0.11	1.24 ± 0.51
**Lactate**	0.35 ± 0.03	0.51 ± 0.07	0.43 ± 0.05	0.30 ± 0.05	0.40 ± 0.05	1.68 ± 0.14	1.02 ± 0.41	1.53 ± 0.51	1.41 ± 0.10	0.92 ± 0.45
**Pyruvate**	0.07 ± 0.01	0.05 ± 0.02	0.28 ± 0.17	0.18 ± 0.04	0.22 ± 0.01	1.88 ± 0.10	0.24 ± 0.04	0.47 ± 0.09	0.64 ± 0.09	0.48 ± 0.24
**Malate**	0.12 ± 0.12	2.73 ± 0.47	<0.01	<0.01	<0.01	<0.01	0.85 ± 0.08	<0.01	0.05 ± 0.03	<0.01
**CO_2**[d]	0	0	0	0	0	0	0	0	0	0
**H_2**	20.20 ± 0.27	N.D.	N.D.	N.D.	19.69 ± 0.22	10.17 ± 0.36	N.D.	N.D.	N.D.	17.76 ± 1.90
**Ex. protein**[e]	4.19 ± 0.11	2.51 ± 0.19	3.39 ± 0.19	4.33 ± 0.14	3.61 ± 0.08	2.13 ± 0.08	1.59 ± 0.17	2.43 ± 0.15	1.99 ± 0.07	1.97 ± 0.27
**Alanine**	0.16 ± 0.01	0.17 ± 0.01	0.17 ± 0.01	0.17 ± 0.01	0.14 ± 0.00	0.24 ± 0.00	0.15 ± 0.01	0.22 ± 0.01	0.20 ± 0.01	0.13 ± 0.01
**Asparagine**	<0.01	<0.01	<0.01	<0.01	<0.01	<0.01	<0.01	<0.01	<0.01	<0.01
**Aspartate**	<0.01	0.06 ± 0.01	0.03 ± 0.00	0.02 ± 0.01	0.03 ± 0.02	0.01 ± 0.00	0.02 ± 0.00	<0.01	0.02 ± 0.00	<0.01
**Glycine**	0.03 ± 0.01	0.03 ± 0.00	0.03 ± 0.00	0.02 ± 0.00	0.02 ± 0.00	0.03 ± 0.00	0.02 ± 0.00	0.02 ± 0.00	0.02 ± 0.00	<0.01
**Glutamate**	0.10 ± 0.01	0.10 ± 0.02	0.08 ± 0.01	0.12 ± 0.01	0.10 ± 0.01	0.22 ± 0.01	0.16 ± 0.01	0.18 ± 0.01	0.31 ± 0.01	0.21 ± 0.02
**Glutamine/arginine**[e]	0.03 ± 0.01	0.08 ± 0.02	0.08 ± 0.03	0.06 ± 0.01	0.02 ± 0.00	0.02 ± 0.01	0.08 ± 0.01	0.10 ± 0.01	0.04 ± 0.00	0.01 ± 0.00
**Histidine**	<0.01	<0.01	<0.01	<0.01	<0.01	<0.01	<0.01	0.01 ± 0.00	<0.01	<0.01
**Isoleucine**	0.08 ± 0.00	0.04 ± 0.00	0.09 ± 0.00	0.10 ± 0.01	0.07 ± 0.00	0.63 ± 0.02	0.23 ± 0.03	0.70 ± 0.15	0.57 ± 0.05	0.15 ± 0.05
**Leucine**	0.05 ± 0.00	0.09 ± 0.01	<0.01	0.04 ± 0.04	0.05 ± 0.00	0.16 ± 0.01	0.14 ± 0.01	0.18 ± 0.00	0.18 ± 0.01	0.07 ± 0.01
**Methionine**	<0.01	<0.01	<0.01	<0.01	<0.01	0.39 ± 0.03	<0.01	0.03 ± 0.03	0.06 ± 0.02	0.01 ± 0.01
**Phenylalanine**	0.09 ± 0.01	0.35 ± 0.05	0.10 ± 0.02	0.08 ± 0.01	0.08 ± 0.00	0.35 ± 0.01	0.36 ± 0.11	0.35 ± 0.04	0.48 ± 0.02	0.39 ± 0.09
**Serine**	0.05 ± 0.01	0.04 ± 0.00	0.05 ± 0.00	0.05 ± 0.00	0.04 ± 0.00	0.04 ± 0.00	0.03 ± 0.00	0.04 ± 0.00	0.04 ± 0.00	0.02 ± 0.01
**Threonine**	0.11 ± 0.01	0.13 ± 0.01	0.12 ± 0.01	0.12 ± 0.01	0.10 ± 0.01	0.12 ± 0.00	0.12 ± 0.01	0.10 ± 0.01	0.17 ± 0.02	0.02 ± 0.01
**Tryptophan**	<0.01	0.03 ± 0.00	<0.01	<0.01	<0.01	<0.01	<0.01	<0.01	<0.01	<0.01
**Tyrosine**	0.07 ± 0.00	0.06 ± 0.01	0.04 ± 0.00	<0.01	<0.01	0.15 ± 0.01	0.09 ± 0.01	0.11 ± 0.01	0.31 ± 0.08	<0.01
**Valine**	0.17 ± 0.01	0.06 ± 0.00	0.25 ± 0.02	0.29 ± 0.01	0.11 ± 0.00	3.37 ± 0.08	0.63 ± 0.14	3.11 ± 1.16	3.27 ± 0.42	0.80 ± 0.47
**Sum of all products**	**90 ± 1**	**73 ± 0**	**72 ± 2**	**70 ± 1**	**86 ± 2**	**80 ± 1**	**71 ± 1**	**68 ± 2**	**68 ± 1**	**76 ± 1**
**Sum of amino acids**	**0.96 ± 0.04**	**1.24 ± 0.05**	**1.04 ± 0.07**	**1.08 ± 0.07**	**0.76 ± 0.03**	**5.71 ± 0.09**	**2.06 ± 0.33**	**5.18 ± 1.33**	**5.67 ± 0.42**	**1.83 ± 0.51**

[a] Data are shown as average ± standard deviation for three biological replicates.

[b] The following elemental degree of reductions were used: C = 4, H = 1, O = -2, N = -3, S = 6.

[c] Based on a molecular weight of 24.66 g mol^{-1} and a degree-of-reduction of 4.30 for the biomass composition $CH_{1.71}O_{0.43}N_{0.20}S_{0.01}$ with 4.32 % ash content (60).

[d] Calculated from fermentation product yields and biomass yield (see Materials and Methods)

[e] Based on a molecular weight of a general protein (see Supplemental Table S2 and Materials and Methods).

[f] Estimated lower value based on a degree of reduction of 18.

N.D. not determined

TABLE S5. Nitrogen recovery on consumed ammonium in chemostats limited on either cellobiose or ammonium at a dilution rate of 0.10 h^{-1}.[a]

% (Nmol/Nmol)	Cellobiose-limitation					Ammonium-limitation				
	Reference strain (LL345)	AVM064 (Δ*ppdk* Δ*malE*::P_{eno}-*pyk*)	AVM003 (Δ*ppdk*)	LL1084 (Δ*nfnAB*)	AG1715 (Δ*gogat*)	Reference strain (LL345)	AVM064 (Δ*ppdk* Δ*malE*::P_{eno}-*pyk*)	AVM003 (Δ*ppdk*)	LL1084 (Δ*nfnAB*)	AG1715 (Δ*gogat*)
**Biomass[b]**	50.45 ± 1.13	49.96 ± 1.52	53.18 ± 1.83	37.10 ± 2.43	39.18 ± 1.10	56.01 ± 1.61	72.80 ± 3.19	62.93 ± 4.45	62.60 ± 1.72	57.36 ± 4.53
**Glucose**	0	0	0	0	0	0	0	0	0	0
**Ethanol**	0	0	0	0	0	0	0	0	0	0
**Acetate**	0	0	0	0	0	0	0	0	0	0
**Formate**	0	0	0	0	0	0	0	0	0	0
**Lactate**	0	0	0	0	0	0	0	0	0	0
**Pyruvate**	0	0	0	0	0	0	0	0	0	0
**Malate**	0	0	0	0	0	0	0	0	0	0
**CO_2[c]**	0	0	0	0	0	0	0	0	0	0
**H_2**	0	0	0	0	0	0	0	0	0	0
**Ex. protein[d]**	16.95 ± 1.09	10.85 ± 0.48	13.03 ± 0.43	13.21 ± 0.59	11.14 ± 0.56	10.97 ± 0.44	10.89 ± 1.08	13.01 ± 1.02	11.47 ± 0.50	8.71 ± 0.56
**Alanine**	0.89 ± 0.05	0.98 ± 0.12	0.93 ± 0.03	0.70 ± 0.06	0.58 ± 0.03	1.66 ± 0.06	1.41 ± 0.10	1.62 ± 0.07	1.56 ± 0.04	0.80 ± 0.13
**Asparagine**	0.04 ± 0.00	0.07 ± 0.01	0.05 ± 0.00	0.01 ± 0.00	0.01 ± 0.00	0.02 ± 0.03	0.03 ± 0.00	0.02 ± 0.00	0.03 ± 0.00	<0.01
**Aspartate**	0.04 ± 0.06	0.32 ± 0.04	0.14 ± 0.01	0.09 ± 0.05	0.11 ± 0.07	0.06 ± 0.01	0.18 ± 0.04	0.05 ± 0.00	0.13 ± 0.02	0.02 ± 0.01
**Glycine**	0.28 ± 0.02	0.35 ± 0.04	0.28 ± 0.03	0.19 ± 0.02	0.16 ± 0.02	0.38 ± 0.01	0.38 ± 0.05	0.35 ± 0.02	0.38 ± 0.03	0.06 ± 0.02
**Glutamate**	0.38 ± 0.02	0.39 ± 0.05	0.29 ± 0.01	0.32 ± 0.04	0.29 ± 0.02	1.01 ± 0.05	0.96 ± 0.05	0.88 ± 0.04	1.62 ± 0.06	0.83 ± 0.12
**Glutamine/arginine[e]**	0.19 ± 0.01	0.60 ± 0.16	0.45 ± 0.03	0.35 ± 0.04	0.12 ± 0.02	0.16 ± 0.07	0.98 ± 0.10	1.00 ± 0.09	0.38 ± 0.04	0.11 ± 0.02
**Histidine**	<0.01	<0.01	<0.01	<0.01	<0.01	0.01 ± 0.00	0.12 ± 0.03	0.19 ± 0.05	0.08 ± 0.01	0.05 ± 0.05
**Isoleucine**	0.17 ± 0.01	0.10 ± 0.01	0.20 ± 0.01	0.17 ± 0.03	0.11 ± 0.00	1.74 ± 0.02	0.85 ± 0.11	2.02 ± 0.39	1.76 ± 0.14	0.38 ± 0.16
**Leucine**	0.11 ± 0.01	0.21 ± 0.03	<0.01	0.07 ± 0.06	0.08 ± 0.00	0.44 ± 0.01	0.53 ± 0.04	0.52 ± 0.01	0.55 ± 0.01	0.17 ± 0.04
**Methionine**	<0.01	<0.01	<0.01	<0.01	0.01 ± 0.02	1.09 ± 0.10	0.03 ± 0.01	0.07 ± 0.09	0.17 ± 0.05	0.04 ± 0.03
**Phenylalanine**	0.15 ± 0.01	0.61 ± 0.05	0.14 ± 0.01	0.09 ± 0.02	0.09 ± 0.01	0.72 ± 0.04	1.01 ± 0.33	0.75 ± 0.11	1.12 ± 0.06	0.69 ± 0.14
**Serine**	0.35 ± 0.04	0.30 ± 0.04	0.30 ± 0.00	0.26 ± 0.02	0.21 ± 0.01	0.33 ± 0.03	0.32 ± 0.01	0.35 ± 0.03	0.39 ± 0.04	0.17 ± 0.06
**Threonine**	0.46 ± 0.03	0.56 ± 0.07	0.45 ± 0.04	0.37 ± 0.04	0.30 ± 0.04	0.63 ± 0.01	0.83 ± 0.10	0.54 ± 0.05	1.01 ± 0.08	0.07 ± 0.06
**Tryptophan**	0.01 ± 0.01	0.09 ± 0.01	0.03 ± 0.00	<0.01	<0.01	<0.01	0.02 ± 0.00	0.02 ± 0.00	<0.01	<0.01
**Tyrosine**	0.13 ± 0.01	0.12 ± 0.01	0.07 ± 0.00	<0.01	<0.01	0.33 ± 0.02	0.26 ± 0.03	0.25 ± 0.02	0.77 ± 0.21	<0.01
**Valine**	0.45 ± 0.01	0.19 ± 0.02	0.66 ± 0.03	0.59 ± 0.04	0.24 ± 0.01	11.71 ± 0.11	2.92 ± 0.68	11.17 ± 3.86	12.69 ± 1.43	2.48 ± 1.68
**Sum of all products**	**71 ± 2**	**66 ± 2**	**47 ± 41**	**54 ± 3**	**53 ± 2**	**87 ± 2**	**95 ± 3**	**96 ± 2**	**97 ± 1**	**72 ± 6**
**Sum of amino acids**	**3.66 ± 0.00**	**4.88 ± 0.49**	**2.66 ± 2.31**	**3.23 ± 0.36**	**2.31 ± 0.21**	**20.28 ± 0.36**	**10.83 ± 1.55**	**19.79 ± 4.23**	**22.65 ± 1.40**	**5.87 ± 2.06**

[a] Data are shown as average ± standard deviation for three biological replicates.
[b] Based on a molecular weight of 24.66 g mol^{-1} for the biomass composition $CH_{1.71}O_{0.43}N_{0.20}S_{0.01}$ with 4.32 % ash content (60).
[c] Calculated from fermentation product yields and biomass yield (see Materials and Methods)
[d] Based on a molecular weight of a general protein (see Supplemental Table S2 and Materials and Methods).
[e] Estimated lower value based on 2 nitrogens per mole.

TABLE S6. Biomass-specific conversion rates in chemostats limited on either cellobiose or ammonium at a dilution rate of 0.10 h^{-1}.[a,b]

	Cellobiose-limitation					Ammonium-limitation				
**Unit: mmol $g_{biomass}^{-1}$ h^{-1}**	**Reference strain (LL345)**	**AVM064 (Δ*ppdk* Δ*malE*::P_{eno}-*pyk*)**	**AVM003 (Δ*ppdk*)**	**LL1084 (Δ*nfnAB*)**	**AG1715 (Δ*gogat*)**	**Reference strain (LL345)**	**AVM064 (Δ*ppdk* Δ*malE*::P_{eno}-*pyk*)**	**AVM003 (Δ*ppdk*)**	**LL1084 (Δ*nfnAB*)**	**AG1715 (Δ*gogat*)**
**Ethanol**	2.12 ± 0.15	3.66 ± 0.11	1.99 ± 0.11	2.12 ± 0.05	1.80 ± 0.14	2.65 ± 0.05	3.94 ± 0.20	2.02 ± 0.13	2.46 ± 0.17	1.63 ± 0.09
**Acetate**	3.03 ± 0.01	1.53 ± 0.08	3.02 ± 0.04	3.04 ± 0.03	2.96 ± 0.04	2.33 ± 0.01	1.54 ± 0.07	2.81 ± 0.18	2.48 ± 0.09	1.77 ± 0.15
**Formate**	0.25 ± 0.08	0.53 ± 0.04	0.20 ± 0.03	0.68 ± 0.02	0.16 ± 0.02	0.37 ± 0.03	0.80 ± 0.09	0.43 ± 0.11	0.31 ± 0.07	0.62 ± 0.24
**Lactate**	0.03 ± 0.00	0.05 ± 0.01	0.04 ± 0.00	0.03 ± 0.00	0.03 ± 0.00	0.17 ± 0.01	0.10 ± 0.04	0.15 ± 0.06	0.14 ± 0.02	0.08 ± 0.04
**Pyruvate**	0.01 ± 0.00	0.01 ± 0.00	0.03 ± 0.02	0.02 ± 0.00	0.02 ± 0.00	0.22 ± 0.01	0.03 ± 0.01	0.05 ± 0.01	0.08 ± 0.01	0.05 ± 0.03
**Malate**	0.01 ± 0.01	0.26 ± 0.05	<0.01	<0.01	<0.01	<0.01	0.09 ± 0.01	<0.01	0.01 ± 0.00	<0.01
**CO_2[c]**	5.21 ± 0.10	4.73 ± 0.19	5.14 ± 0.10	4.81 ± 0.08	4.93 ± 0.09	5.15 ± 0.04	4.97 ± 0.17	4.94 ± 0.18	5.16 ± 0.23	3.13 ± 0.33
**H_2**	10.6 ± 0.3	N.D.	N.D.	N.D.	10.2 ± 0.2	6.1 ± 0.3	N.D.	N.D.	N.D.	9.0 ± 1.1
**Ex. protein[d]**	0.20 ± 0.01	0.13 ± 0.01	0.16 ± 0.01	0.21 ± 0.01	0.17 ± 0.00	0.12 ± 0.00	0.09 ± 0.01	0.13 ± 0.00	0.11 ± 0.01	0.09 ± 0.01
**Total amino acids[e]**	0.06 ± 0.00	0.07 ± 0.01	0.06 ± 0.00	0.07 ± 0.00	0.05 ± 0.00	0.29 ± 0.01	0.11 ± 0.02	0.26 ± 0.08	0.28 ± 0.02	0.08 ± 0.02
**Unit: µmol $g_{biomass}^{-1}$ h^{-1}**										
**Alanine**	14.2 ± 0.5	15.8 ± 1.4	13.9 ± 1.1	15.0 ± 0.5	12.0 ± 0.2	23.7 ± 0.3	15.6 ± 1.3	21.6 ± 1.9	19.6 ± 0.9	11.3 ± 1.1
**Asparagine**	0.3 ± 0.0	0.6 ± 0.1	0.3 ± 0.1	<0.2	<0.2	<0.2	<0.2	<0.2	<0.2	<0.2
**Aspartate**	0.6 ± 1.1	5.2 ± 0.7	2.1 ± 0.2	2.0 ± 1.0	2.3 ± 1.5	0.8 ± 0.1	2.0 ± 0.5	0.6 ± 0.0	1.7 ± 0.2	0.3 ± 0.2
**Cysteine**	-80 ± 1	-304 ± 11	-324 ± 5	-335 ± 11	-73 ± 54	-262 ± 117	-409 ± 20	-404 ± 128	-473 ± 21	-66 ± 29
**Glycine**	4.5 ± 0.5	5.7 ± 0.7	4.4 ± 0.3	4.1 ± 0.1	3.2 ± 0.4	5.4 ± 0.1	4.1 ± 0.5	4.7 ± 0.6	4.8 ± 0.4	0.8 ± 0.4
**Glutamate**	6.1 ± 0.2	6.3 ± 1.0	4.6 ± 0.1	6.9 ± 0.5	5.9 ± 0.3	14.4 ± 0.3	10.5 ± 0.4	11.8 ± 0.9	20.4 ± 0.7	11.6 ± 1.4
**Glutamine/arginine[f]**	1.5 ± 0.1	4.8 ± 1.1	4.3 ± 1.3	3.7 ± 0.4	1.3 ± 0.1	1.2 ± 0.5	5.4 ± 0.6	6.6 ± 0.8	2.4 ± 0.3	0.8 ± 0.1
**Histidine**	<0.2	<0.2	<0.2	<0.2	<0.2	<0.2	0.5 ± 0.1	0.8 ± 0.2	0.3 ± 0.0	<0.2
**Isoleucine**	2.8 ± 0.1	1.5 ± 0.1	3.1 ± 0.1	3.6 ± 0.5	2.3 ± 0.0	24.9 ± 0.7	9.3 ± 1.2	27.1 ± 7.1	22.2 ± 2.0	5.2 ± 1.8
**Leucine**	1.8 ± 0.0	3.4 ± 0.3	<0.2	1.5 ± 1.3	1.6 ± 0.0	6.2 ± 0.2	5.8 ± 0.5	7.0 ± 0.4	6.9 ± 0.2	2.3 ± 0.4
**Methionine**	<0.2	<0.2	<0.2	<0.2	<0.2	15.6 ± 1.3	0.3 ± 0.1	1.0 ± 1.2	2.2 ± 0.6	0.5 ± 0.3
**Phenylalanine**	2.4 ± 0.2	9.9 ± 1.2	2.4 ± 0.5	2.0 ± 0.4	2.0 ± 0.1	10.3 ± 0.2	11.1 ± 3.4	10.0 ± 1.5	14.2 ± 1.1	9.8 ± 1.8
**Serine**	5.7 ± 0.5	4.9 ± 0.5	4.7 ± 0.2	5.5 ± 0.2	4.3 ± 0.2	4.8 ± 0.4	3.5 ± 0.3	4.7 ± 0.1	5.0 ± 0.4	2.4 ± 0.9
**Threonine**	7.3 ± 0.5	9.0 ± 1.1	7.3 ± 0.7	7.8 ± 0.5	6.2 ± 0.7	9.1 ± 0.3	9.2 ± 1.1	7.2 ± 1.2	12.7 ± 1.0	1.0 ± 0.7
**Tryptophan**	<0.2	0.8 ± 0.1	<0.2	<0.2	<0.2	<0.2	<0.2	<0.2	<0.2	<0.2
**Tyrosine**	2.0 ± 0.1	1.9 ± 0.2	1.1 ± 0.1	<0.2	<0.2	4.7 ± 0.2	2.9 ± 0.3	3.3 ± 0.1	9.8 ± 2.7	<0.2
**Valine**	7.2 ± 0.3	3.0 ± 0.2	10.5 ± 0.2	12.7 ± 0.5	4.9 ± 0.2	167.4 ± 5.1	32.1 ± 7.1	150.8 ± 63.0	159.8 ± 19.1	33.9 ± 20.5

[a] Data are shown as average ± standard deviation for three biological replicates.
[b] Negative values represent consumption and positive values represent production
[c] Calculated from fermentation product yields and biomass yield (see Materials and Methods)
[d] Based on a molecular weight of a general protein (see Supplemental Table S2 and Materials and Methods).
[e] Excluding cysteine.
[f] Sum of the two amino acids.
N.D. not determined.

TABLE S7. Concentrations of substrates and products in chemostats limited on either cellobiose or ammonium at a dilution rate of 0.10 h^{-1}.[a,b]

	Cellobiose-limitation					Ammonium-limitation				
	**Reference strain (LL345)**	**AVM064 (Δ*ppdk* Δ*malE*::P_{eno}-*pyk*)**	**AVM003 (Δ*ppdk*)**	**LL1084 (Δ*nfnAB*)**	**AG1715 (Δ*gogat*)**	**Reference strain (LL345)**	**AVM064 (Δ*ppdk* Δ*malE*::P_{eno}-*pyk*)**	**AVM003 (Δ*ppdk*)**	**LL1084 (Δ*nfnAB*)**	**AG1715 (Δ*gogat*)**
**OD (at 600 nm)**	1.38 ± 0.03	1.28 ± 0.01	1.39 ± 0.02	1.41 ± 0.01	1.42 ± 0.03	0.83 ± 0.01	0.90 ± 0.01	1.22 ± 0.11	1.02 ± 0.04	0.87 ± 0.08
**Cell dry weight (g L^{-1})**	0.63 ± 0.01	0.62 ± 0.03	0.69 ± 0.02	0.63 ± 0.00	0.66 ± 0.01[e]	0.33 ± 0.01	0.44 ± 0.02	0.38 ± 0.03	0.38 ± 0.01	0.35 ± 0.03[e]
**Cb in feed (mmol L^{-1})**	13.72 ± 0.09	14.55 ± 0.08	13.90 ± 0.23	14.22 ± 0.06	14.02 ± 0.12	13.71 ± 0.10	14.57 ± 0.01	13.54 ± 0.11	14.18 ± 0.06	13.93 ± 0.16
**Residual cb (mmol L^{-1})**	<0.01	<0.01	<0.01	<0.01	<0.01	5.34 ± 0.08	3.21 ± 0.11	4.85 ± 0.31	4.60 ± 0.30	6.53 ± 0.88
**Glu in feed (mmol L^{-1})**	38.16 ± 0.05	39.41 ± 0.94	37.36 ± 1.08	38.79 ± 0.69	39.03 ± 0.58	4.81 ± 0.14	4.92 ± 0.05	4.85 ± 0.03	4.93 ± 0.04	4.93 ± 0.05
**Residual glu (mmol L^{-1})**	28.04 ± 0.47	29.44 ± 0.42	27.51 ± 0.64	25.07 ± 0.79	24.84 ± 0.51	<0.01	<0.01	0.03 ± 0.00	<0.01	<0.01
**NH_4^+ in feed (mmol L^{-1})**	0.07 ± 0.01	<0.01	0.04 ± 0.01	<0.01	<0.01	0.08 ± 0.00	<0.01	0.04 ± 0.00	<0.01	<0.01
**Residual NH_4^+ (mmol L^{-1})**	0.03 ± 0.01	0.02 ± 0.00	0.02 ± 0.02	<0.01	0.02 ± 0.01	0.18 ± 0.01	0.23 ± 0.04	0.31 ± 0.05	0.27 ± 0.03	0.23 ± 0.04
**Ethanol (mmol L^{-1})**	11.28 ± 0.71	19.08 ± 0.19	11.35 ± 0.43	11.56 ± 0.14	9.98 ± 0.84	7.55 ± 0.29	14.91 ± 0.42	6.23 ± 0.25	8.12 ± 0.41	4.87 ± 0.68
**Acetate (mmol L^{-1})**	18.97 ± 0.25	9.44 ± 0.22	20.24 ± 0.30	19.63 ± 0.26	19.35 ± 0.06	7.84 ± 0.15	6.88 ± 0.16	10.21 ± 1.23	9.69 ± 0.16	6.19 ± 0.11
**Formate (mmol L^{-1})**	1.58 ± 0.52	3.27 ± 0.30	1.37 ± 0.17	4.37 ± 0.11	1.04 ± 0.12	1.24 ± 0.10	3.59 ± 0.34	1.59 ± 0.46	1.20 ± 0.28	2.20 ± 0.87
**Lactate (mmol L^{-1})**	0.19 ± 0.02	0.30 ± 0.04	0.24 ± 0.02	0.17 ± 0.03	0.23 ± 0.03	0.56 ± 0.05	0.46 ± 0.19	0.53 ± 0.17	0.54 ± 0.05	0.28 ± 0.17
**Pyruvate (mmol L^{-1})**	0.05 ± 0.00	0.04 ± 0.01	0.18 ± 0.11	0.12 ± 0.02	0.15 ± 0.01	0.75 ± 0.03	0.13 ± 0.02	0.20 ± 0.04	0.29 ± 0.04	0.18 ± 0.11
**Malate (mmol L^{-1})**	0.06 ± 0.06	1.59 ± 0.28	<0.01	<0.01	<0.01	<0.01	0.38 ± 0.03	<0.01	0.02 ± 0.01	<0.01
**H_2 (mol%)**	1.00 ± 0.03	<0.01	<0.01	<0.01	1.03 ± 0.02	0.31 ± 0.01	<0.01	<0.01	<0.01	0.49 ± 0.05
**Ex. protein (mg mL^{-1})**	0.144 ± 0.004	0.092 ± 0.007	0.118 ± 0.006	0.155 ± 0.005	0.127 ± 0.003	0.045 ± 0.002	0.045 ± 0.004	0.053 ± 0.004	0.048 ± 0.002	0.036 ± 0.002
**Ex. protein (mmol L^{-1})[d]**	1.28 ± 0.04	0.81 ± 0.06	1.05 ± 0.05	1.37 ± 0.04	1.13 ± 0.03	0.40 ± 0.02	0.40 ± 0.04	0.47 ± 0.04	0.43 ± 0.02	0.32 ± 0.02
**Alanine (µmol L^{-1})**	89.3 ± 3.0	97.0 ± 6.1	92.9 ± 6.8	97.0 ± 3.6	77.5 ± 0.3	79.8 ± 0.6	69.5 ± 4.2	77.9 ± 3.7	76.7 ± 1.7	38.9 ± 6.8
**Asparagine (µmol L^{-1})**	1.9 ± 0.1	3.5 ± 0.5	2.3 ± 0.4	0.9 ± 0.1	0.7 ± 0.1	0.5 ± 0.7	0.8 ± 0.1	0.4 ± 0.1	0.7 ± 0.1	0.3 ± 0.3
**Aspartate (µmol L^{-1})**	3.9 ± 6.8	32.2 ± 3.7	14.3 ± 1.6	12.9 ± 6.5	14.1 ± 8.5	2.8 ± 0.4	8.8 ± 1.9	2.2 ± 0.1	6.6 ± 1.1	0.5 ± 0.3
**Cys in feed (µmol L^{-1})**	775 ± 18	2455 ± 70	2760 ± 49	2744 ± 39	859 ± 351	1188 ± 424	2262 ± 77	2221 ± 392	2487 ± 15	760 ± 54
**Residual cys (µmol L^{-1})**	270 ± 12	583 ± 67	587 ± 31	582 ± 17	432 ± 38	303 ± 16	433 ± 39	775 ± 83	638 ± 18	685 ± 33
**Glycine (µmol L^{-1})**	28.0 ± 3.0	35.2 ± 3.3	29.8 ± 2.7	26.5 ± 0.7	20.5 ± 2.0	18.1 ± 0.6	18.4 ± 2.2	16.8 ± 1.2	18.7 ± 1.3	2.7 ± 0.7
**Glutamate (µmol L^{-1})**	38.1 ± 1.0	38.7 ± 5.6	30.7 ± 1.4	44.4 ± 3.1	33.6 ± 2.6	48.5 ± 1.8	47.1 ± 2.3	42.6 ± 2.0	79.9 ± 3.7	32.2 ± 5.6
**Gln/arginine (µmol L^{-1})[e]**	9.6 ± 0.5	29.5 ± 6.3	28.6 ± 9.4	23.8 ± 2.4	9.1 ± 1.6	3.9 ± 1.8	24.2 ± 2.3	24.0 ± 2.1	9.5 ± 1.1	5.5 ± 3.9
**Histidine (µmol L^{-1})**	<0.1	<0.1	<0.1	<0.1	<0.1	0.2 ± 0.1	2.0 ± 0.4	3.0 ± 0.7	1.2 ± 0.1	0.7 ± 0.6
**Isoleucine (µmol L^{-1})**	17.5 ± 0.5	9.5 ± 0.6	20.5 ± 0.9	23.0 ± 2.8	14.8 ± 0.2	83.6 ± 1.5	41.8 ± 5.3	97.1 ± 19.0	86.7 ± 6.2	18.4 ± 7.7
**Leucine (µmol L^{-1})**	11.2 ± 0.2	21.0 ± 1.6	<0.1	9.8 ± 8.5	10.5 ± 0.3	20.9 ± 0.7	25.9 ± 1.9	25.2 ± 0.5	26.9 ± 0.6	8.1 ± 1.9
**Methionine (µmol L^{-1})**	0.6 ± 1.0	0.3 ± 0.5	<0.1	<0.1	1.4 ± 2.5	52.5 ± 3.6	1.5 ± 0.3	3.5 ± 4.4	8.6 ± 2.6	6.1 ± 1.7
**Phenylalanine (µmol L^{-1})**	14.8 ± 1.2	60.8 ± 8.3	16.2 ± 3.6	12.8 ± 2.3	12.7 ± 0.6	34.8 ± 1.2	49.7 ± 15.7	36.1 ± 5.5	55.3 ± 2.5	32.8 ± 6.1
**Serine (µmol L^{-1})**	35.6 ± 3.2	30.2 ± 2.1	31.7 ± 0.3	35.5 ± 1.4	27.7 ± 0.6	16.0 ± 1.7	15.7 ± 0.6	17.1 ± 1.3	19.4 ± 1.9	7.4 ± 2.3
**Threonine (µmol L^{-1})**	46.1 ± 3.3	55.5 ± 5.2	48.9 ± 5.4	50.6 ± 3.2	39.5 ± 3.4	30.5 ± 0.6	41.0 ± 4.9	25.9 ± 2.6	49.8 ± 3.6	3.7 ± 2.3
**Tryptophan (µmol L^{-1})**	0.7 ± 0.1	4.7 ± 0.6	1.2 ± 0.7	0.5 ± 0.1	<0.1	<0.1	0.4 ± 0.1	0.4 ± 0.1	<0.1	<0.1
**Tyrosine (µmol L^{-1})**	12.5 ± 0.2	11.5 ± 1.1	7.2 ± 0.3	<0.1	<0.1	15.7 ± 0.4	12.8 ± 1.3	11.8 ± 1.1	38.2 ± 10.4	<0.1
**Valine (µmol L^{-1})**	45.4 ± 1.7	18.4 ± 1.1	70.3 ± 3.2	82.1 ± 4.2	32.0 ± 1.5	563 ± 12	144 ± 32	538 ± 188	625 ± 67	123 ± 83

[a] Data are shown as average ± standard deviation for three biological replicates.

[b] Abbreviations: cb, cellobiose; glu, glucose; cys, cysteine; gln, glutamine.

[c] Based on OD with an average CDW-to-OD conversion factor of 2.16 (OD/CDW) in cellobiose limitation and 2.48 in nitrogen limitation.

[d] Based on a molecular weight of a general protein (see Supplemental Table S2 and Materials and Methods).

[e] Sum of the two amino acids.

TABLE S8. Lactate dehydrogenase activities for different strains in this study at different conditions, expressed in µmol $mg_{protein}^{-1}$ min^{-1}.[a]

Strain	Exponential phase in batch	Cellobiose-limitation	Ammonium-limitation
**WT**	0.51 ± 0.03	N.D.	N.D.
**AVM003 (*Δppdk*)**	0.30 ± 0.06	N.D.	N.D.
**AVM064 (*Δppdk Δme*::P_{eno}-*pyk*)**	0.33 ± 0.03	N.D.	N.D.
**LL345 (*Δhpt*)**	N.D.	0.69 ± 0.02	2.65 ± 0.26
**AG1715 (*Δhpt Δgogat)***	N.D.	0.40 ± 0.02	0.9 ± 0.03

[a] Data are shown as average ± standard deviation for four technical replicates of biological duplicates.

N.D. not determined
